# Hepatocellular carcinoma risk stratification to identify patients suitable for intensive surveillance in viral hepatitis: the SELECT score

**DOI:** 10.1007/s00330-025-12060-8

**Published:** 2025-10-25

**Authors:** Yeun-Yoon Kim, Won Chang, Jeong Min Lee, Se Woo Kim, Jae Seok Bae, Jeongin Yoo, Sun Kyung Jeon, HeeSoo Kim, Young Hoon Kim, Jin-Young Choi, Eun Ju Cho, Yun Bin Lee, Sook-Hyang Jeong, Do Young Kim, Yunhee Choi, Jeong Hee Yoon

**Affiliations:** 1 Department of Radiology and Research Institute of Radiological Science, Severance Hospital, Yonsei University College of Medicine, Seoul, Republic of Korea; 2 Department of Radiology, Seoul National University Bundang Hospital, Seongnam, Republic of Korea; 3 Department of Radiology, Seoul National University College of Medicine, Seoul, Republic of Korea; 4 Department of Radiology, Seoul National University Hospital, Seoul, Republic of Korea; 5 Institute of Radiation Medicine, Seoul National University Medical Research Center, Seoul, Republic of Korea; 6 Department of Internal Medicine and Liver Research Institute, Seoul National University Hospital and College of Medicine, Seoul, Republic of Korea; 7 Department of Internal Medicine and Liver Research Institute, Seoul National University Bundang Hospital, Seongnam, Republic of Korea; 8 Department of Internal Medicine, Seoul National University College of Medicine, Seoul, Republic of Korea; 9 Department of Internal Medicine and Institute of Gastroenterology, Yonsei University College of Medicine, Seoul, Republic of Korea; 10 Yonsei Liver Center, Severance Hospital, Seoul, Republic of Korea; 11 Division of Biostatistics, Medical Research Collaborating Center, Seoul National University Hospital, Seoul, Republic of Korea; 12 Present Address: Department of Radiology and Center for Imaging Sciences, Samsung Medical Center, Sungkyunkwan University School of Medicine, Seoul, Republic of Korea

**Keywords:** Hepatocellular carcinoma, Cirrhosis, Hepatitis B, Hepatitis C, Ultrasound

## Abstract

**Objective:**

A risk-stratification strategy can improve the effectiveness of intensive hepatocellular carcinoma (HCC) surveillance with an alternative modality. However, such strategies and prediction models incorporating ultrasound features remain undeveloped for a suitable population. Therefore, we developed and validated an HCC risk prediction model using ultrasound features in patients with viral hepatitis who are potentially eligible for intensive surveillance.

**Materials and methods:**

This retrospective multicenter study included 17,557 HCC-naïve patients with viral hepatitis who underwent US surveillance between 2005 and 2015. In the development dataset (*n* = 7918), clinical and US features were analyzed to establish the prediction model. Factors associated with HCC were identified by multivariable Cox regression analysis. Model performance was compared to existing prediction models in internal (*n* = 3393) and external (*n* = 6246) validation datasets.

**Results:**

The SELECT model included age, male sex, diabetes, serum albumin and alanine aminotransferase levels, platelet count, and ultrasound-detected cirrhosis and multiple cirrhotic nodules. In the external validation dataset, the low-, intermediate-, and high-risk groups had 0.8%, 6.9%, and 16.1% 5-year cumulative HCC incidence, respectively. In those with an estimated annual HCC incidence ≥ 2.5% (SELECT score > −2.04), the 5-year cumulative HCC incidence was 15.5%. The SELECT model had better discrimination capability than aMAP, THRI, ADRESS-HCC, the Velazquez score, and mPAGE-B (Uno C-index, 0.791 vs. 0.740, 0.668, 0.658, 0.650, and 0.740, respectively; all adjusted *p* < 0.001).

**Conclusion:**

The SELECT model better estimated HCC risk than other models in viral hepatitis patients. Intensive surveillance with alternative modalities may be considered based on this model.

**Key Points:**

***Question*** *Ultrasound features have not been incorporated into hepatocellular carcinoma risk prediction models, despite ultrasound being the primary surveillance modality.*

***Findings*** *The SELECT model, incorporating demographics, laboratory findings and ultrasound features (cirrhosis and multiple cirrhotic nodules), demonstrated superior performance compared to existing models.*

***Clinical relevance*** *The SELECT model effectively identifies viral hepatitis patients with ≥ 2.5% annual HCC risk who would benefit from intensive surveillance using alternative imaging modalities, optimizing resource allocation and achieving higher diagnostic yield.*

**Graphical Abstract:**

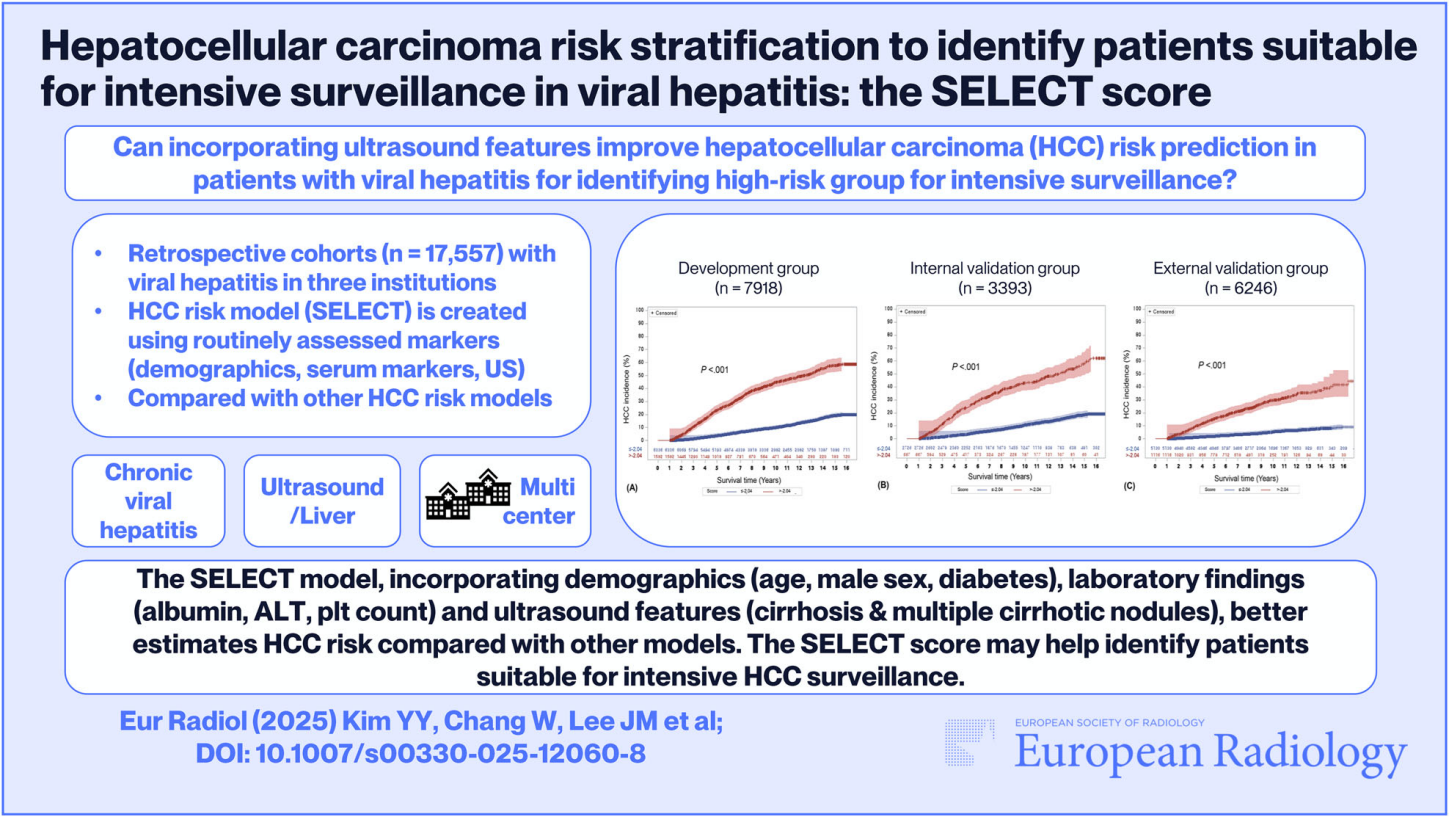

## Introduction

Primary liver cancer, with hepatocellular carcinoma (HCC) accounting for 75–85% of cases, is the third leading cause of cancer-related deaths globally [1]. The high mortality rate is largely due to the advanced stage of the disease at diagnosis; therefore, surveillance is recommended for patients at risk of HCC to facilitate early detection and improve clinical outcomes [2]. Although ultrasound (US) has been a primary surveillance modality, it has shown suboptimal sensitivity for detecting early-stage HCC [3–5].

Recent studies have reported the usefulness of surveillance using alternative imaging modalities for detecting early-stage HCCs [3–5]. However, due to the limited accessibility and high costs associated with intensive HCC surveillance using these alternative modalities, their use should be selective, targeting patients at higher risk of developing HCC to achieve an acceptable diagnostic yield (DY) and cost-effectiveness [6, 7]. HCC risk prediction models were developed based on clinical and laboratory parameters [8–12] and rarely incorporated imaging features that may reflect HCC risk and the real-time feasibility of US-based surveillance. Recognizing this gap, we aimed to develop and validate a model to predict HCC risk using US features in patients with viral hepatitis who are potentially eligible for intensive HCC surveillance with alternative modalities.

## Materials and methods

This retrospective multicenter study was conducted after the approval of the institutional review boards at Seoul National University Hospital (institution 1), Seoul National University Bundang Hospital (institution 2), and Severance Hospital (institution 3). The requirement for informed consent was waived due to the retrospective study design.

### Study sample

We reviewed the electronic medical records from institution 1 to identify patients who underwent biannual HCC surveillance using US from January 2005 to December 2015. Eligible patients met the following criteria at the time of the index US (i.e., the earliest US exam with complete laboratory data): (1) chronic hepatitis B or C; (2) aged between 40 and 75 years; and (3) follow-up period ≥ 1 year after index US. The exclusion criteria included: (1) prior diagnosis or treatment of HCC; (2) Child-Pugh classification B or C; (3) lack of blood tests (complete blood count [CBC] and liver function test [LFT]) within 8 weeks of the US; (4) unknown HCC development status (no diagnostic test performed for US-detected observations); and (5) unavailable US images.

The same eligibility criteria were applied to identify patients at institutions 2 and 3 during the study period from January 2008 to December 2015.

For all patients, the following data were collected on age, sex, diabetes status, body mass index, history of alcohol intake, and results of serologic tests, including CBC, LFT, prothrombin time (PT), prothrombin activity, and alpha-fetoprotein (AFP) within 8 weeks of the index US. Additionally, the last clinical visit, development of HCC (Supplementary Material), and use of antiviral medication were recorded.

### Analysis of index US reports

The US findings were consistently described across these institutions. The reports specifically noted the presence of the following features: (1) coarse or cirrhotic echotexture of the liver parenchyma, (2) hepatic steatosis, (3) cirrhotic nodules (characterized as scattered subcentimeter hypo- or hyperechoic nodules), (4) ascites, and (5) splenomegaly. If a report did not mention a feature, it was assumed to be absent.

### HCC risk model development and validation

For model development, patients from institution 1 were randomly divided into development or internal validation datasets at a 7:3 ratio. Data from institutions 2 and 3 were used as an external validation dataset. For each patient, imaging features from the index US, clinical information at the time of the index US, and serologic tests conducted within 8 weeks of the index US were analyzed. Detailed statistical methods for model development and validation are outlined in the Supplementary Material.

### Calculation of HCC risk with existing risk stratification systems

With the collected data, we calculated the risk scores using five established HCC scoring systems: aMAP, Toronto HCC Risk Index (THRI), ADRESS-HCC, the scoring system endorsed by Velazquez et al (the Velazquez score), and modified PAGE-B (mPAGE-B) [8–12]. For each system, we applied the reported cut-off values to identify the high-risk group: ≥ 60 for aMAP, > 240 for THRI, ≥ 4.71 for ADRESS-HCC, > 2.33 for the Velazquez score, and ≥ 13 for mPAGE-B. Detailed information about each scoring system is provided in the Supplementary Material.

### Inter-observer agreement for significant US features

To assess the inter-observer agreement for significant US features included in the model, patients who underwent an index US examination from July to December 2015 were consecutively selected from the institution 1 cohort. Four fellowship-trained body radiologists (J.S.B., J.Y., S.K.J., and H.K., with 7, 6, 6, and 2 years of post-fellowship experience, respectively) independently reviewed the index US. They recorded the presence of US features in a binary manner (Supplementary Material).

### Statistical analysis

The demographics of the three datasets were compared using either analysis of variance or the Kruskal–Wallis test for continuous variables, and the chi-square test for categorical variables. The cumulative incidence of HCC after the index US was estimated using the Kaplan–Meier method. Univariable and multivariable Cox proportional hazards regression analyses were conducted to identify factors associated with incident HCC and to predict the high-risk group. And the final selected model included predictors significant on multivariable Cox regression. All possible two-way interactions among the selected predictors were systematically evaluated, and statistically significant interactions were retained in the final model (Supplementary Material). The model’s discrimination capability was evaluated using Uno C-index and calibration slopes, being assessed with a calibration plot. X-tile plots were utilized to determine two statistically optimal cut-offs, categorizing patients into low-, intermediate-, and high-risk groups (Supplementary Material). To identify patients eligible for intensive surveillance, the cut-off was selected to target patients with an estimated annual HCC incidence ≥ 2.5% [6, 7]. Uno C-indexes, sensitivities, and specificities for predicting 5-year incident HCC were calculated and compared with existing HCC risk scoring systems. Inter-observer agreement for US features was assessed using Fleiss kappa statistics: poor, < 0.20; fair, 0.20–0.39; moderate, 0.40–0.59; substantial, 0.60–0.79; and almost perfect, > 0.80 [13]. Statistical analysis was performed using SAS (version 9.4, SAS Institute) and R software (version 4.3.0, R Foundation). Adjusted *p*-value of < 0.05 was considered statistically significant.

## Results

### Baseline characteristics

**Fig. 1:**
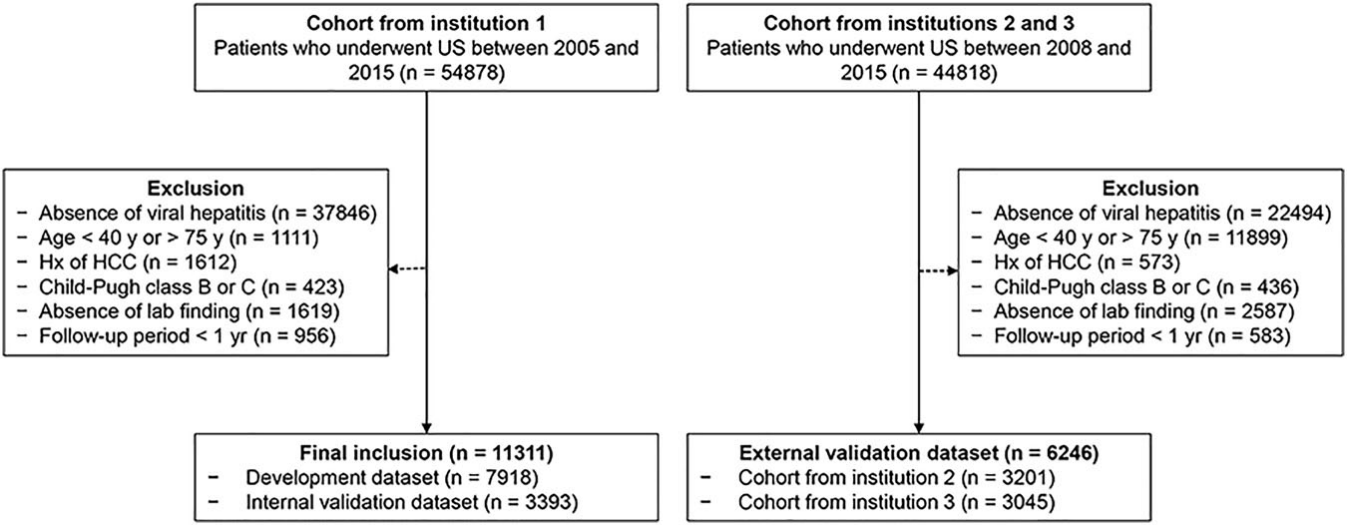
Study flow. HCC, hepatocellular carcinoma; US, ultrasound. Institution 1 = Seoul National University Hospital, Institution 2 = Seoul National University Bundang Hospital, Institution 3 = Severance Hospital

A total of 17,557 patients (9558 men; mean age, 53.4 ± 8.8 years) were included from three institutions. Of these, 11,311 patients from institution 1 were randomly assigned to either the development (*n* = 7918) or the internal validation (*n* = 3393) datasets. The remaining 6246 patients from the other two institutions were allocated to the external validation dataset (Fig. 1). The median follow-up durations were 8.9, 8.9, and 8.1 years for the development, internal, and external validation datasets, respectively. The cumulative incidence of HCC was 1362, 577, and 494 during the follow-up for the three datasets, respectively. The characteristics of each dataset are described in Table 1 and the Supplementary Material.

**Table 1:** Baseline characteristics

Variable		Development (*n* = 7918)	Internal validation (*n* = 3393)	External validation (*n* = 6246)	*p*-value
Age (years)		53.4 (53.2, 53.6)	53.2 (52.9, 53.5)	53.5 (53.3, 53.7)	0.36
Female		54.9 (54.6, 55.2)	54.6 (54.1, 55)	54.6 (54.3, 54.9)	0.30
Male		52.2 (51.9, 52.5)	52.1 (51.7, 52.5)	52.5 (52.2, 52.8)	0.20
Sex (male)		55.9 (4425)	55.6 (1885)	52.0 (3248)	< 0.001
Etiology					
HBV		82.6 (6541)	81.6 (2769)	81.8 (5112)	< 0.001
HCV		16.2 (1279)	16.6 (563)	17.7 (1105)	
Co-infection		1.2 (98)	1.8 (61)	0.5 (29)	
Antiviral agent (yes)		16.7 (1324)	18.1 (613)	7.7 (478)	< 0.001
Alcohol intake (yes)		2.5 (196)	2.4 (79)	4.0 (127)	< 0.001
Diabetes mellitus (yes)		23.4 (1855)	22.6 (768)	18.0 (1124)	< 0.001
BMI classification (kg/m^2^)*	Underweight (< 18.5)	2.2 (173)	2.0 (46)	2.3 (127)	0.64
	Normal (18.5–22.9)	32.4 (2558)	32.0 (750)	32.6 (1808)	
	Overweight (23–24.9)	51.2 (4034)	51.5 (1205)	60.0 (3326)	
	Obesity (≥ 25)	14.2 (1122)	14.5 (340)	4.1 (229)	
Albumin (g/dL)		4.3 (2.8, 5.3)	4.3 (2.8, 5.2)	4.3 (2.8, 5.7)	< 0.001
Total bilirubin (mg/dL)		0.9 (0.2, 3.0)	0.9 (0.2, 3.0)	0.8 (0.1, 3.0)	< 0.001
AST (U/L)		29 (6, 686)	30 (7, 540)	28 (6, 967)	< 0.001
ALT (U/L)		30 (3, 1624)	29 (2, 757)	27 (5, 1601)	< 0.001
PT (INR)		1.00 (0.82, 2.27)	1.00 (0.83, 2.15)	1.01 (0.76, 2.29)	< 0.001
Platelet (10^9^/L)		178 (7, 1305)	176 (8, 453)	181 (1, 891)	< 0.001
AFP (ng/mL)		5 (1, 1833)	5 (1, 820)	2.76 (0.3, 8153.3)	< 0.001
US features					
Liver parenchyma					
Normal echo CLD Cirrhosis		32.3 (2555) 40.6 (3218) 27.1 (2145)	29.1 (986) 43.9 (1491) 27.0 (916)	37.4 (2337) 38.3 (2392) 24.3 (1517)	< 0.001
Hepatic steatosis (yes)		29.3 (2321)	28.8 (978)	15.6 (974)	< 0.001
Cirrhotic nodules					
None Single or a few Multiple		86.3 (6832) 5.0 (396) 8.71 (690)	86.7 (2941) 4.3 (148) 9.0 (304)	86.2 (5386) 1.3 (83) 12.4 (777)	< 0.001
Splenomegaly (yes)		18.5 (1462)	19.5 (660)	15.3 (488)	< 0.001
Ascites (yes)		0 (0)	0 (0)	0.1 (7)	
HCC risk scoring system					
aMAP score		64.4 (64.2, 64.6)	64.5 (64.3, 64.7)	63.8 (63.7, 64.0)	< 0.001
THRI		206.7 (205.3, 208.2)	207.7 (205.5, 209.9)	169.9 (168.1, 171.8)	< 0.001
HCC-ADRESS		5.22 (5.21, 5.23)	5.21 (5.19, 5.23)	5.19 (5.18, 5.21)	0.01
Velazquez score		1.2 (1.2, 1.2)	1.2 (1.2, 1.2)	1.8 (1.8, 1.8)	< 0.001
Modified PAGE-B		11 (10.9, 11)	11 (10.9, 11.1)	10.8 (10.7, 10.9)	< 0.001

Values are mean (95% CIs), median (IQR) or percentage (number). A *p*-value < 0.05 indicates a significant difference between the three groups

*HBV* hepatitis B virus, *HCV* hepatitis C virus, *BMI* body mass index, *AST* aspartate aminotransferase, *ALT* alanine aminotransferase, *PT* prothrombin time, *INR* international normalized ratio, *AFP* alpha-fetoprotein, *US* ultrasound, *CLD* chronic liver disease, *HCC* hepatocellular carcinoma, *THRI* Toronto HCC Risk Index

* Unavailable in 2237, 1052, and 756 patients in development, internal, and external validation datasets

### Factors associated with incident HCC

In the development dataset, the cumulative incidence of HCC at 1-, 5-, 10-, and 15-year follow-up intervals was 0.01%, 7.59%, 16.97%, and 27.08%, respectively.

All significant variables in univariable analysis (Table 2) were included in the multivariable analysis. In multivariable analysis, two US features, cirrhosis and cirrhotic nodules, were categorized in a binary manner to minimize the effect of inter-observer variability in clinical practice (e.g., cirrhosis vs. non-cirrhosis; multiple cirrhotic nodules vs. no, single, or a few nodules). The multivariable regression analysis revealed that age, male sex, diabetes, low albumin levels, low platelet count, high alanine aminotransferase (ALT) levels, and the presence of cirrhosis and multiple cirrhotic nodules were significantly associated with HCC development (Table 2). The hazard ratio (HR) for US-defined cirrhosis was higher in patients with normal platelet counts (3.228 [95% CI: 2.744, 3.798]) compared to those with low platelet counts (1.770, [95% CI: 1.512, 2.072]), with a statistically significant interaction between platelet count and cirrhosis on US (Fig. S1, *p* < 0.001) (Supplementary Material).

**Table 2:** Factors associated with HCC development in the development dataset

Variables	Category	Univariable HR (95% CI)	*p*-value	Multivariable HR (95% CI)	*p*-value
Age (years)		1.025 (1.019, 1.031)	< 0.001	1.028 (1.021, 1.034)	< 0.001
Sex	Female	Ref		Ref	
	Male	1.568 (1.401, 1.755)	< 0.001	1.714 (1.524, 1.926)	< 0.001
Etiology	HBV	Ref			
	HCV	1.027 (0.885, 1.192)	0.73		
	Co-infection of HBV and HCV	1.638 (1.119, 2.4)	0.01		
Alcohol intake	No/denial	Ref			
	Yes	1.726 (1.304, 2.283)	0.001		
Diabetes mellitus	No	Ref		Ref	
	Yes	1.352 (1.204, 1.518)	< 0.001	1.153 (1.025, 1.296)	0.02
BMI classification* (kg/m^2^)	Underweight (< 18.5)	Ref			
	Normal (18.5–22.9)	1.217 (0.784, 1.888)	0.38		
	Overweight (23–24.9)	1.425 (0.923, 2.199)	0.11		
	Obesity (≥ 25)	1.404 (0.894, 2.206)	0.14		
Albumin (g/dL)		0.261 (0.228, 0.298)	< 0.001	0.429 (0.370, 0.497)	< 0.001
Total bilirubin (mg/dL)		1.828 (1.638, 2.04)	< 0.001		
Platelet (10^9^/L)^†^		0.989 (0.989, 0.99)	< 0.001		
	Cirrhosis on US (no)			0.992 (0.991, 0.994)	< 0.001
	Cirrhosis on US (yes)			0.998 (0.997, 0.999)	< 0.001
AST (U/L)		1.004 (1.003, 1.005)	< 0.001		
ALT (U/L)		1.001 (1.001, 1.002)	< 0.001	1.001 (1.000, 1.002)	0.006
PT (INR)		6.644 (5.01, 8.812)	< 0.001		
Liver parenchyma on US	Normal echo	Ref			
	CLD	1.621 (1.364, 1.926)	< 0.001		
	Cirrhosis	5.557 (4.744, 6.508)	< 0.001		
Liver parenchyma on US (binary classification)	Normal echo or CLD	Ref			
	Cirrhosis	4.125 (3.705, 4.592)	< 0.001		
Cirrhosis on US^†‡^	Platelet count = 100 (per 10^9^/L)			1.770 (1.512, 2.072)	< 0.001
	Platelet count = 150 (per 10^9^/L)			2.390 (2.100, 2.715)	< 0.001
	Platelet count = 200 (per 10^9^/L)			3.228 (2.744, 3.798)	< 0.001
Hepatic steatosis on US	Absence	Ref			
	Presence	0.531 (0.463, 0.609)	< 0.001		
Cirrhotic nodules on US	None	1			
	Single/a few	2.758 (2.325, 3.273)	< 0.001		
	Multiple	3.516 (3.072, 4.024)	< 0.001		
Cirrhotic nodules on US (binary classification)	None or single/a few	Ref		Ref	
	Multiple	3.204 (2.805, 3.659)	< 0.001	1.398 (1.206, 1.620)	< 0.001
Splenomegaly on US	Absence	Ref			
	Presence	2.677 (2.395, 2.992)	< 0.001		

Multivariable Cox regression analysis was performed. *p*-value < 0.05 indicates statistical significance

*HCC* hepatocellular carcinoma, *HR* hazard ratio, *HBV* hepatitis B virus, *HCV* hepatitis C virus, *BMI* body mass index, *AST* aspartate aminotransferase, *ALT* alanine aminotransferase, *PT* prothrombin time, *INR* international normalized ratio, *US* ultrasound, *CLD* chronic liver disease

^*^ Unavailable in 2372 patients

^†^ Values are presented conditionally due to a significant interaction (*p* < 0.001) between platelet count and cirrhosis on US

^‡^ In this analysis, specific platelet counts (100, 150, and 200 × 10^9^/L) corresponding to low, low normal limit, and normal values were used as reference points for assessing the interaction with US-defined cirrhosis

### Development and validation of SELECT scoring system

#### Model development

The SELECT risk score was named after the significant factors on multivariable Cox regression (**S**ex, ag**E,** **L**iver function test, diab**E**tes, platelet **C**ount, and **T**rans-abdominal ultrasound findings), where the interaction between platelet count and US cirrhosis was significant (*p* < 0.001) among all possible two-way interactions. The inclusion of US features significantly improved model performance compared to a clinical model without US features in internal and external validation datasets (Tables S1–2, Fig. S2). The Cox proportional hazards model was constructed as follows:

$${{{\rm{SELECT}}}} \; {{{\rm{score}}}}\;= 	 \; 0.54\times {{{\rm{male\; sex}}}}+0.027\times {{{\rm{age}}}}+0.001\times {{{\rm{ALT}}}} \\ 	 -0.847\times {{{\rm{albumin}}}}+0.14\times {{{\rm{diabetes}}}}\left({{{\rm{yes}}}}\right) \\ 	 -0.008\times {{{\rm{platelet}}}}\; {{{\rm{count}}}}+0.006 \\ 	 \times {{{\rm{cirrhosis}}}}\; {{{\rm{on}}}}\; {{{\rm{US}}}}\left({{{\rm{yes}}}}\right)\times \left({{{\rm{platelet}}}}\; {{{\rm{count}}}}-5\right) \\ 	 +0.34\times {{{\rm{multiple}}}}\; {{{\rm{cirrhotic}}}}\; {{{\rm{nodules}}}}\; {{{\rm{on}}}}\; {{{\rm{US}}}}\left({{{\rm{yes}}}}\right)$$

where age is in years, ALT in U/L, albumin in g/dL, and platelet count in 10^3^/mm^3^.

The estimate of 5-year cumulative incidence of HCC was calculated as follows:

$$1-{0.359}^{\exp ({{{\rm{SELECT}}}}\; {{{\rm{score}}}})}.$$

**Fig. 2:**
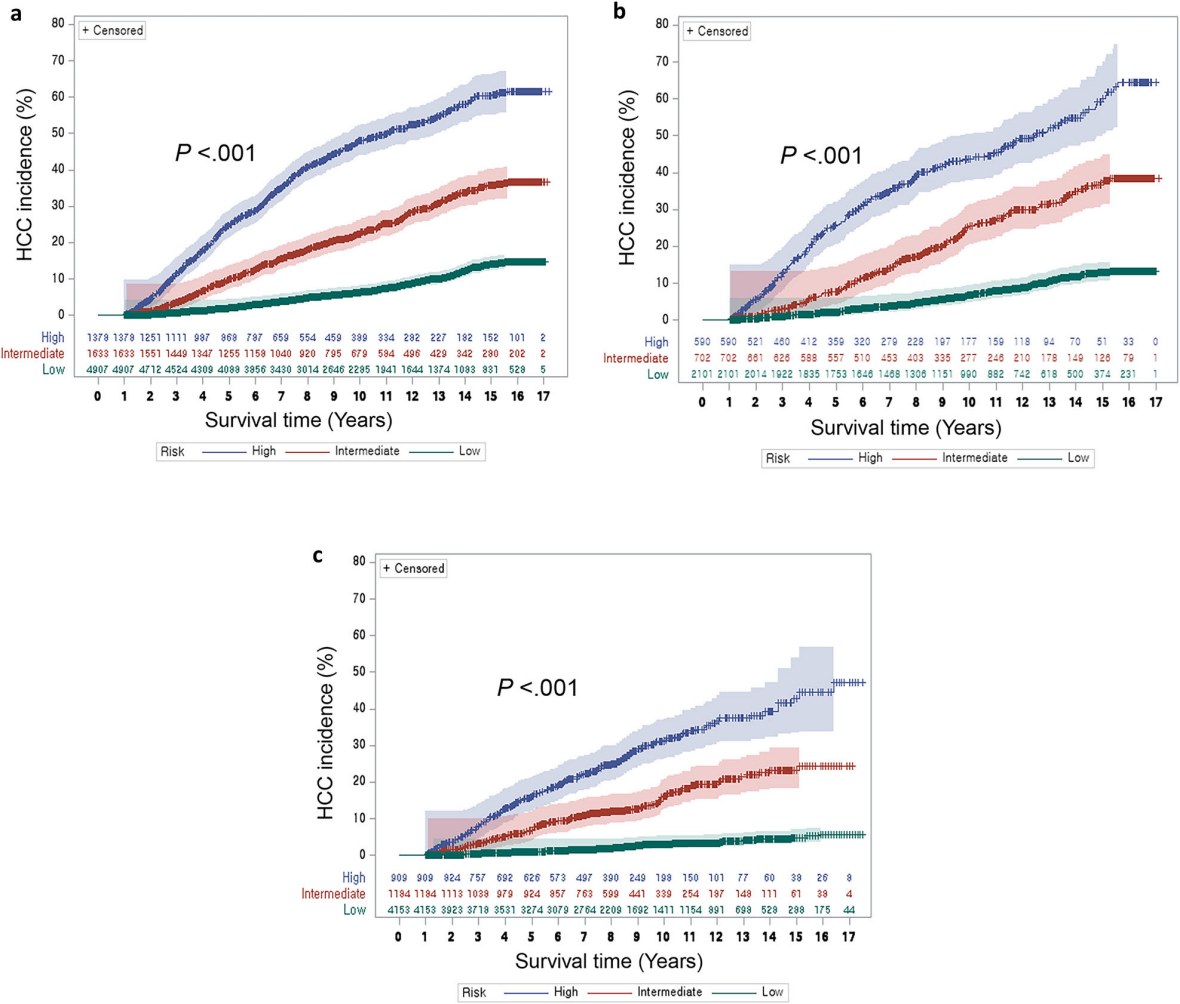
Cumulative incidence of HCC according to the SELECT risk stratification. Cumulative hepatocellular carcinoma (HCC) incidence according to the SELECT score in (**a**) development, (**b**) internal validation, and (**c**) external validation datasets (blue: high risk, red: intermediate risk, green: low risk)

In development dataset, x-tile plots demonstrated two cut-off values of the SELECT score (≤ −2.720 and > 1.941) to stratify patients into low-, intermediate- and high-risk groups, and 62% (4907/7918), 21% (1633/7918) and 17% (1378/7918) of patients were assigned to each group (Table S3). The 5-year cumulative incidence of HCC was 2.06%, 9.98%, 24.83% in low-, intermediate- and high-risk groups, respectively (Table S3, Figs. 2a, S3, S4).

#### Internal validation

With the two cut-offs (≤ −2.720 and > 1.941), patients were assigned to low- (61.92%, 2101/3393), intermediate- (20.68%, 702/3393) and high-risk groups (17.39%, 590/3393) (Table S3). The 5-year cumulative incidence of HCC was 2.17%, 7.95%, and 25.97% in the low-, intermediate-, and high-risk groups, respectively (Table S3, Fig. 2b). Compared to the low-risk group, intermediate- and high-risk groups showed HRs of 3.566 (95% CI: 2.869, 4.432) and 8.117 (95% CI: 6.640, 9.922).

#### External validation

In this dataset, 66.5% (4153/6246), 19.0% (1184/6246), and 14.6% (909/6246) of patients were assigned to the low-, intermediate-, and high-risk groups, respectively. The 5-year cumulative incidence of HCC was 0.80%, 6.90%, and 16.10% in each group (Table S3, Fig. 2c). The HRs for the intermediate- and high-risk groups were 5.930 (95% CI: 4.617, 7.616) and 13.038 (95% CI: 10.313, 16.484).

### Selection of eligible patients for intensive HCC surveillance using alternative modalities

**Fig. 3:**
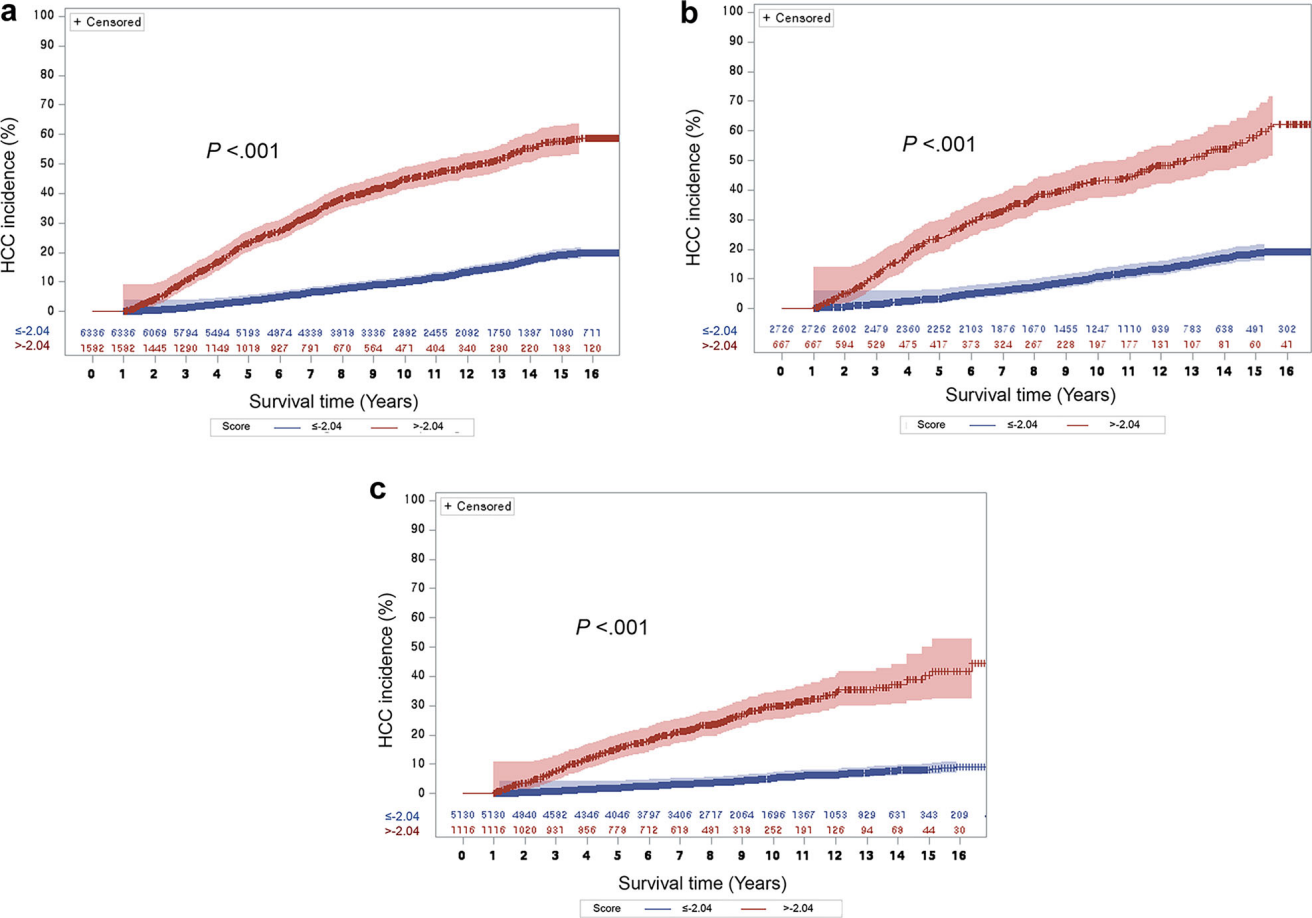
Cumulative incidence of HCC according to a SELECT cut-off of > −2.04. Cumulative hepatocellular carcinoma (HCC) incidence for the high-risk group based on SELECT score in (**a**) development, (**b**) internal validation, and (**c**) external validation datasets (red: SELECT score > −2.04, blue: SELECT score ≤ −2.04)

In the development dataset, after the prediction model was finalized, a post hoc cut-off value of > −2.04 was selected. This threshold corresponded to an estimated 5-year HCC incidence of 12.5%, assuming a constant annual HCC incidence of ≥ 2.5%. Based on this cut-off, 20.0% (1582/7918) of patients belong to this group (Table 3). The 5-year cumulative incidence of HCC was estimated as 23.5% (Fig. 3a).

**Table 3:** Discrimination capability of the SELECT scoring system

Variables	Development dataset (*n* = 7918)	Internal validation dataset (*n* = 3393)	External validation dataset (*n* = 6246)
Number of patients with SELECT score > −2.04	20.0 (1582)	19.7 (667)	17.9 (1116)
5-year HCC cumulative incidence (%)*	23.5 [21.3, 25.7]	24.1 [20.7, 27.5]	15.5 [13.2, 17.7]
10-year HCC cumulative incidence (%)*	45.0 [42.1, 47.8]	40.3 [36.1, 44.5]	29.8 [26.4, 33.1]
Hazard ratio^†^	4.949 [4.449, 5.505]	5.048 [4.284, 5.948]	6.707 [5.614, 8.012]
Sensitivity (%)^‡^	61.2 (336/549) [57.1, 65.3]	63.0 (144/228) [57.0, 69.0]	64.0 (156/244) [58.0, 70.0]
Specificity (%)^‡^	83.1 (6123/7369) [82.2, 84.0]	83.0 (2641/3156) [82.0, 85.0]	84.0 (5042/6002) [83.0, 85.0]
Positive predictive value (%)^‡^	21.2 (336/1582) [19.3, 23.3]	21.56 (144/668) [18.5, 24.9]	14.0 (156/1116) [12, 16.2]
Negative predictive value (%)^‡^	96.6 (6123/6336) [96.2, 97.1]	96.9 (2641/2725) [96.2, 97.5]	98.3 (5042/5130) [97.9, 98.6]
Accuracy (%)^‡^	81.6 (6459/7918) [80.7, 82.4]	82.1 (2785/3393) [80.8, 83.4]	83.2 (5198/6246) [82.3, 84.1]

Values are percentages (absolute numbers or numerators/denominators) unless otherwise specified. Numbers in brackets are 95% CI

*HCC* hepatocellular carcinoma

* Incidence in patients with SELECT score > −2.04

^†^ Hazard ratio of SELECT score > −2.04 compared to that of score ≤ −2.04

^‡^ For 5-year HCC development

In the internal validation dataset, the SELECT cut-off identified 19.7% (667/3393) of patients as the eligible population, in which the 5- and 10-year cumulative HCC incidence rates were 24.1% and 40.3%, respectively (Table 3, Fig. 3b). In the external validation dataset, 17.9% (1116/6246) of patients were identified as being eligible for alternative surveillance, and their 5- and 10-year cumulative HCC incidence rates were 15.5% (95% CI: 13.2, 17.7) and 29.8% (95% CI: 26.4, 33.1) (Table 3, Fig. 3c). Those with SELECT score ≤ −2.04, the 5- and 10-year cumulative incidence of HCC were 3.4% and 10.8% in internal validation dataset, and 1.9% and 5.4% in external validation dataset (Table S4). The Uno C-index was 0.747 (95% CI: 0.726, 0.767) and 0.791 (95% CI: 0.764, 0.819) in the internal and external validation datasets, respectively. The calibration slope was 1.020 (95% CI: 0.920, 1.110) and 1.165 (95% CI: 1.067, 1.262) in the internal and external validations (Fig. S5).

### Comparison of model performance with other risk scoring systems

**Fig. 4:**
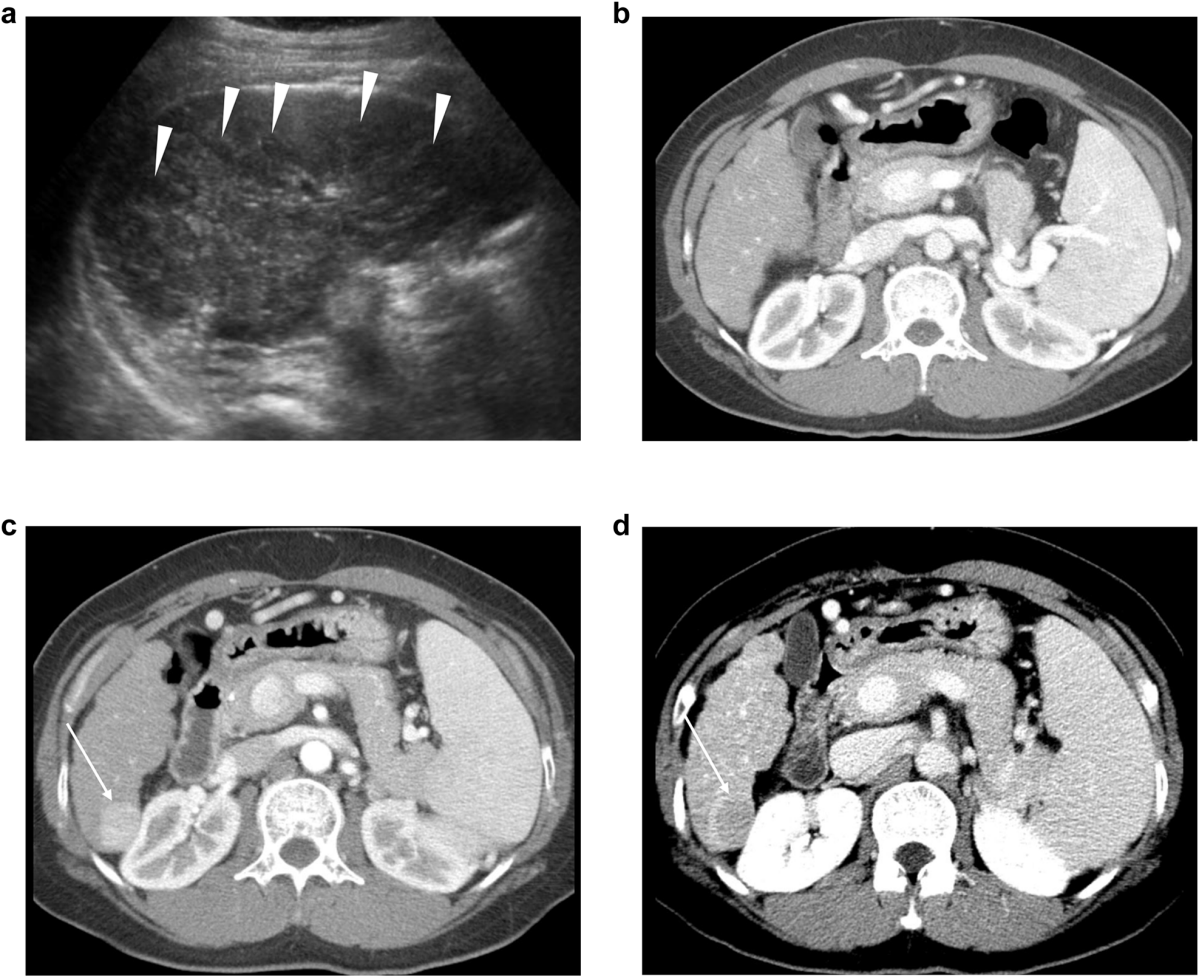
A 49-year-old non-diabetic woman with hepatitis B deemed to be high-risk by SELECT (a score of −1.93). **a** An index ultrasound (US) image shows cirrhotic liver parenchyma and multiple hypo- and hyperechoic cirrhotic nodules (arrowheads). On 5-month follow-up, liver CT confirms the absence of hepatocellular carcinoma (HCC) at index US (**b**). The patient developed HCC at an 11-month follow-up, which shows arterial phase hyperenhancement (**c**) and delayed washout (**d**, arrows). While aMAP (a score of 68.49) also classified the patients as high-risk, the other models of THRI (236), ADRESS-HCC (4.64), Velazquez score (1.66), and mPAGE-B (10) failed to classify the patients as high-risk

In external validation dataset, the SELECT score showed an Uno C-index of 0.791 (95% CI: 0.761, 0.822), which was higher than those of aMAP (0.740), THRI (0.668), ADRESS-HCC (0.658), the Velazquez score (0.650) (*p* < 0.001 for all) (Table 4, Fig. 4). The SELECT score exhibited better performance than mPAGE-B in hepatitis B patients (0.790 [95% CI: 0.758, 0.823] vs. 0.732 [0.703, 0.761], *p* < 0.001) (Table 4).

**Table 4:** Performance of risk scoring systems in the external validation dataset

Risk scoring system	Total (*n* = 6246)		Hepatitis B viral infection (*n* = 5112)	
	Uno C-index	*p*-value	Uno C-index	*p*-value
SELECT	0.791 [0.761, 0.822]	Reference	0.790 [0.758, 0.823]	Reference
aMAP	0.740 [0.710, 0.770]	< 0.001	0.735 [0.698, 0.772]	< 0.001
THRI	0.668 [0.632, 0.704]	< 0.001	0.669 [0.620, 0.718]	< 0.001
ADRESS-HCC	0.658 [0.628, 0.688]	< 0.001	0.657 [0.623, 0.691]	< 0.001
Velazquez score	0.650 [0.627, 0.674]	< 0.001	0.648 [0.614, 0.682]	< 0.001
mPAGE-B	0.740 [0.714, 0.766]	< 0.001	0.732 [0.703, 0.761]	< 0.001

Values in brackets are 95% CI

The C-index of each system was compared with that of SELECT, and the *p*-values were adjusted using the step-down Bonferroni method

*THRI* Toronto HCC Risk Index

The SELECT score (> −2.04) identified 17.9% (1116/6246) of patients as a risk group eligible for alternative surveillance, and it had a sensitivity of 64.0% and a specificity of 84.0% for predicting 5-year HCC occurrence (Table 5). Both aMAP and ADRESS-HCC scores classified 72.0% (4499/6246) and 82.5% (5155/6246) as eligible for alternative surveillance, which resulted in higher sensitivities (95.0%) but low specificities (< 30%). THRI and Velazquez score revealed higher specificities than the SELECT score (80.0% and 92.0%, respectively), but their sensitivities were < 40%. mPAGE-B classified 25.1% (1565/6246) of patients as eligible for intensive surveillance and had a similar sensitivity to the SELECT score (62.0%, *p* = 0.54) but lower specificity in all patients (76.0%, *p* < 0.001) and in hepatitis B subgroup (84.0% [95% CI: 83.0, 85.0] vs. 78.0% [77.0, 80.0], *p* < 0.001) (Table S5).

**Table 5:** Performance of risk scores for predicting HCC development in the external validation dataset

Variables (*n* = 6246)	SELECT	aMAP	THRI	ADRESS-HCC	Velazquez et al	mPAGE-B
Cut-offs	> −2.04	≥ 60	> 240	≥ 4.71	> 2.33	≥ 13
Number of patients*	17.9 (1116)	72.0 (4499)	20.3 (1268)	82.5 (5155)	9.1 (567)	25.1 (1565)
5-year HCC cumulative incidence (%)	15.5 [13.2, 17.7]	5.7 [5, 6.4]	7.5 [6, 9]	5 [4.4, 5.6]	15.4 [12.3, 18.5]	10.8 [9.2, 12.4]
10-year HCC cumulative incidence (%)	29.8 [26.4, 33.1]	12.5 [11.3, 13.7]	16.42 [14, 18.9]	11.23 [10.1, 12.3]	27.88 [23.3, 32.5]	23.06 [20.3, 25.8]
Sensitivity (%)^†^	64.0 (156/244) [58.0, 70.0]	95.0 (233/244) [92.0, 98.0]	36.0 (87/244) [30.0, 42.0]	95.0 (233/244) [92.0, 98.0]	32.0 (79/244) [27.0, 39.0]	62.0 (151/244) [55.0, 68.0]
*p*-value^‡^	Ref	< 0.001	< 0.001	< 0.001	< 0.001	0.54
Specificity (%)^†^	84.0 (5042/6002) [83.0, 85.0]	29.0 (1736/6002) [28.0, 30.0]	80.0 (4821/6002) [79.0, 81.0]	18.0 (1080/6002) [17.0, 19.0]	92.0 (5514/6002) [91.0, 93.0]	76.0 (4588/6002) [75.0, 78.0]
*p*-value^‡^	Ref	< 0.001	< 0.001	< 0.001	< 0.001	< 0.001
PPV (%)^†^	14.0 (156/1116) [12, 16.2]	5.2 (233/4499) [0.3, 1.1]	6.9 (87/1268) [5.5, 8.4]	4.5 (233/5155) [4, 5.1]	13.9 (79/567) [11.2, 17.1]	9.7 (151/1565) [8.2, 11.2]
*p*-value^‡^	Ref	< 0.001	< 0.001	< 0.001	0.97	< 0.001
NPV (%)^†^	98.3 (5042/5130) [97.9, 98.6]	99.4 (1736/1747) [98.9, 99.7]	96.9 (4821/4978) [96.3, 97.3]	99.0 (1080/1091) [98.2, 99.5]	97.1 (5514/5679) [96.6, 97.5]	98.0 (4588/4681) [97.6, 98.4]
*p*-value^‡^	Ref	0.002	< 0.001	0.15	< 0.001	0.15
Accuracy (%)^†^	83.2 (5198/6246) [82.3, 84.1]	31.5 (1969/6246) [30.4, 32.7]	78.6 (4908/6246) [77.5, 79.6]	21.0 (1313/6246) [20, 22.1]	89.6 (5593/6246) [88.8, 90.3]	75.9 (4739/6246) [74.8, 76.9]
*p*-value^‡^	Ref	< 0.001	< 0.001	< 0.001	< 0.001	< 0.001

Values are percentages (absolute numbers or numerators/denominators). Numbers in brackets are 95% CI

*HCC* hepatocellular carcinoma, *THRI* Toronto HCC Risk Index, *PPV* positive predictive value, *NPV* negative predictive value

* Number of patients with values equal to or higher than the cut-offs

^†^ for 5-year HCC development

^‡^ adjusted *p*-value using the step-down Bonferroni method for comparison with SELECT

### Inter-observer agreement for significant US features

A total of 217 patients were included in this analysis, and their demographics are summarized in the Supplementary Material. The inter-observer agreement among the four readers was substantial (κ = 0.64 [95% CI: 0.55, 0.71]) for cirrhosis (Fig. S6) and moderate (κ = 0.56 [95% CI: 0.46, 0.67]) for multiple cirrhotic nodules (Fig. S7).

## Discussion

We developed SELECT model for stratifying the risk of developing HCC in viral hepatitis patients without hepatic decompensation. This model incorporates routinely assessed factors such as demographics, laboratory findings, and US features to maximize its applicability during surveillance. For patients eligible for intensive surveillance (SELECT score > −2.04), the 5-year cumulative incidence of HCC was 15.5% in the external validation dataset, with superior discrimination capability compared to other risk scoring systems. Therefore, we believe that the SELECT model is able to identify an eligible group for intensive surveillance.

SELECT model is unique because it incorporates US features, whereas previous models have relied solely on demographic and laboratory data [8–10, 12, 14]. Including US features is justified, given their wide availability as a primary surveillance modality and their ability to provide critical information on liver disease status. Indeed, the study results showed that the predictive impact of cirrhosis on US became more pronounced in patients with normal platelet counts, suggesting that US-defined cirrhosis serves as a more potent risk factor in the absence of clinically significant portal hypertension. This finding underscores the added value of incorporating US features into the risk model, as it captures risk differences even in patients with seemingly less advanced liver disease. Also, the presence of multiple cirrhotic nodules on US was independently associated with an increased risk of HCC, which aligns with recent findings that US features are associated with the development of HCC [15]. Although a few studies have reported that subcentimeter nodules do not necessarily confer a higher risk [16], a recent systematic review found that up to 21.3% of patients with subcentimeter nodules developed HCC at the patient level [17]. Also, the presence of multiple cirrhotic nodules has been linked to false negative results in surveillance US, which compromises the performance of US and supports the use of alternative surveillance modalities [18]. One may argue that the presence of cirrhotic nodules is subjective and an indication of diagnostic examinations. However, the presence of subcentimeter nodules does not mandate immediate diagnostic examinations or intensive US [19]. Additionally, by employing binary classification, moderate to substantial inter-observer agreement for US features was achieved, making it readily applicable in daily clinical practice.

In our univariable analysis, hepatic steatosis was paradoxically associated with a reduced risk of HCC. This finding appears to be confounded by the severity of underlying liver disease. In our data, non-cirrhotic patients more frequently presented hepatic steatosis compared with cirrhotic patients, which was consistent with recent study results [20]. Therefore, in the unadjusted analysis, non-steatotic patients included an overrepresented number of high-risk cirrhotic patients, which may have led to an inverse association between steatosis and HCC risk. This likely explains its exclusion from the final SELECT model, which prioritizes more direct and consistent predictors of HCC. This finding also informs the ongoing debate in the literature [21, 22], where the impact of steatosis may be influenced by diagnostic methods and the presence of metabolic dysfunction [23–31]. Further studies are warranted to better define the utility of steatosis for HCC risk stratification in viral hepatitis patients.

The study population was restricted to patients with adequate life expectancy and without hepatic decompensation, suitable candidates for long-term intensive surveillance. In this population, the SELECT model with a cut-off of > −2.04 demonstrated 64.0% sensitivity and 84.0% specificity for predicting 5-year HCC development in the external dataset. While aMAP and ADRESS-HCC showed high sensitivity, they classified most patients (72.0–82.5%) as requiring intensive surveillance, potentially increasing healthcare burden and reducing DY. Conversely, THRI and Velazquez score exhibited low sensitivity (32–36%), potentially excluding many patients who might benefit from intensive surveillance. Although the Velazquez score achieved the highest overall accuracy (89.6%) due to high specificity (92.0%), this high accuracy is misleading, given the unacceptably low sensitivity for a screening tool. The SELECT model better balances sensitivity and specificity for identifying appropriate surveillance candidates in this specific population, whereas other models were developed with cut-offs for general high-risk groups, including older patients and those with decompensated cirrhosis who are unsuitable for intensive surveillance.

Of note, we selected a cut-off (> −2.04) aimed at achieving an annual incidence of ≥ 2.5%. This threshold is lower than those used in previous prospective studies, which reported incidences of 5–15% [3, 4]. However, we believe this cut-off is appropriate in the era of antiviral agents with reduced HCC incidence, which ranged from 0.5 to 2.1 per 100 person-years [32, 33]. A few recent studies also reported lower cut-offs for cost-effectiveness in alternative surveillance, though cut-offs varied depending on countries [6, 7]. Additionally, the criteria for the surveillance population may not be highly specific; applying overly stringent selection criteria targeting 5–15% could result in the exclusion of patients who might benefit from alternative surveillance.

The actual incidence of HCC was lower in the external validation group compared with that in the development and internal validation datasets. While the precise cause remains unclear, the intensive surveillance practices in South Korea might provide an explanation. According to national health insurance data, South Korea had already implemented intensive surveillance using alternative imaging modalities during the study period, exceeding international guidelines [34]. Therefore, we hypothesize that the intensity of surveillance varies based on institutional triage and physicians’ preferences, which in turn influences the demographics observed in US surveillance. Despite these variations, our SELECT model demonstrated strong discrimination capability and performance in the external validation dataset.

There are several limitations to the study. First, selection bias was inevitable due to its retrospective nature. Although the association between clinical conditions such as obesity and HCC development is documented, our analysis did not prove it. We attribute this to limitations inherent in a retrospective study design, including the presence of missing data (30%). Future studies are therefore warranted to clarify the role of other clinical conditions in improving the model’s predictive performance. Second, we did not consider known risk factors such as viral genotypes or viral titers. This was a deliberate choice, as the primary goal was to develop a model that could be immediately implemented in daily practice, to identify the population eligible for “intensive surveillance.” Third, elastography was not included in the model as we intended to use routinely assessed variables. Moreover, liver stiffness can normalize in patients receiving antiviral therapy, yet guidelines still diagnose cirrhosis based on grayscale imaging and clinical criteria even in the presence of normal stiffness values [35]. Therefore, while we acknowledge that elastography may offer additional insights, our reliance on US criteria for diagnosing cirrhosis remains clinically appropriate. Additional studies incorporating liver stiffness could be valuable. Fourth, while our study included both chronic liver disease and cirrhosis, this approach aligns with several validated risk prediction models, such as aMAP and mPAGE-B [8, 12]. Although these conditions represent different stages of disease progression, combining them in a single predictive model offers practical advantages, particularly in cases where the distinction between advanced fibrosis and early cirrhosis is not clear-cut in patients without decompensation. Fifth, we excluded nonviral cirrhosis from the study to maximize the DY of SELECT model, and further research is necessary to validate the model for nonviral cirrhosis, including metabolic dysfunction-associated steatotic liver disease and alcoholic liver diseases. Sixth, we could not stratify model performance by US visualization quality, as the US-LI-RADS visualization scoring system was not available during the study period. While we believe the SELECT model remains applicable across visualization categories, given that the two key US features are often readily detected even with suboptimal visualization, future prospective validation stratified by visualization scores would strengthen the model’s clinical applicability. Lastly, as our study included patients over an extended period, the treatments received varied across time.

In conclusion, the SELECT model, which integrates routinely assessed clinical and surveillance US features, proved effective in distinguishing individuals who have an estimated annual HCC incidence of ≥ 2.5% among those with viral hepatitis, and it may offer better risk stratification for effective HCC surveillance.

## Supplementary information

Supplementary information

## Data Availability

Individual patient data will not be publicly shared due to privacy concerns. Data generated or analyzed during the study are available from the corresponding author upon request.

## Abbreviations

AFP: Alpha-fetoprotein
ALT: Alanine aminotransferase
DY: Diagnostic yield
HCC: Hepatocellular carcinoma
US: Ultrasound

## References

[1] Sung H, Ferlay J, Siegel RL et al (2021) Global cancer statistics 2020: GLOBOCAN estimates of incidence and mortality worldwide for 36 cancers in 185 countries. CA Cancer J Clin 71:209–249. 10.3322/caac.21660 33538338 10.3322/caac.21660

[2] Zhang B-H, Yang B-H, Tang Z-Y (2004) Randomized controlled trial of screening for hepatocellular carcinoma. J Cancer Res Clin Oncol 130:417–422. 10.1007/s00432-004-0552-0 15042359 10.1007/s00432-004-0552-0 PMC12161851

[3] Kim SY, An J, Lim Y-S et al (2017) MRI with liver-specific contrast for surveillance of patients with cirrhosis at high risk of hepatocellular carcinoma. JAMA Oncol 3:456–463. 10.1001/jamaoncol.2016.3147 27657493 10.1001/jamaoncol.2016.3147 PMC5470420

[4] Yoon JH, Lee JM, Lee DH et al (2020) A comparison of biannual two-phase low-dose liver CT and US for HCC surveillance in a group at high risk of HCC development. Liver Cancer 9:503–517. 10.1159/000506834 33083277 10.1159/000506834 PMC7548851

[5] Kim DH, Yoon JH, Choi MH et al (2024) Comparison of non-contrast abbreviated MRI and ultrasound as surveillance modalities for HCC. J Hepatol 81:461–470. 10.1016/j.jhep.2024.03.048 38636849 10.1016/j.jhep.2024.03.048

[6] Kim H-L, An J, Park J-A et al (2019) Magnetic resonance imaging is cost-effective for hepatocellular carcinoma surveillance in high-risk patients with cirrhosis. Hepatology 69:1599–1613. 10.1002/hep.30330 30365164 10.1002/hep.30330

[7] Decharatanachart P, Pan-Ngum W, Peeraphatdit T et al (2024) Cost-utility analysis of non-contrast abbreviated magnetic resonance imaging for hepatocellular carcinoma surveillance in cirrhosis. Gut Liver 18:135–146. 10.5009/gnl230089 37560799 10.5009/gnl230089 PMC10791494

[8] Fan R, Papatheodoridis G, Sun J et al (2020) aMAP risk score predicts hepatocellular carcinoma development in patients with chronic hepatitis. J Hepatol 73:1368–1378. 10.1016/j.jhep.2020.07.025 32707225 10.1016/j.jhep.2020.07.025

[9] Sharma SA, Kowgier M, Hansen BE et al (2018) Toronto HCC risk index: a validated scoring system to predict 10-year risk of HCC in patients with cirrhosis. J Hepatol 68:92–99. 10.1016/j.jhep.2017.07.033 10.1016/j.jhep.2017.07.033 28844936

[10] Flemming JA, Yang JD, Vittinghoff E, Kim WR, Terrault NA (2014) Risk prediction of hepatocellular carcinoma in patients with cirrhosis: the ADRESS-HCC risk model. Cancer 120:3485–3493. 10.1002/cncr.28832 10.1002/cncr.28832 PMC4553222 25042049

[11] Velázquez RF, Rodríguez M, Navascués CA et al (2003) Prospective analysis of risk factors for hepatocellular carcinoma in patients with liver cirrhosis. Hepatology 37:520–527. 10.1053/jhep.2003.50093 12601348 10.1053/jhep.2003.50093

[12] Kim JH, Kim YD, Lee M et al (2018) Modified PAGE-B score predicts the risk of hepatocellular carcinoma in Asians with chronic hepatitis B on antiviral therapy. J Hepatol 69:1066–1073. 10.1016/j.jhep.2018.07.018 30075230 10.1016/j.jhep.2018.07.018

[13] Viera AJ, Garrett JM (2005) Understanding interobserver agreement: the kappa statistic. Fam Med 37:360–363 15883903

[14] Lee HW, Ahn SH (2016) Prediction models of hepatocellular carcinoma development in chronic hepatitis B patients. World J Gastroenterol 22:8314–8321. 10.3748/wjg.v22.i37.8314 27729738 10.3748/wjg.v22.i37.8314 PMC5055862

[15] Park MK, Lee DH, Hur BY et al (2023) Effectiveness of US surveillance of hepatocellular carcinoma in chronic hepatitis B: US LI-RADS visualization score. Radiology 307:e222106. 10.1148/radiol.222106 37249427 10.1148/radiol.222106

[16] Singal AG, Ghaziani TT, Mehta N et al (2023) Recall patterns and risk of primary liver cancer for subcentimeter ultrasound liver observations: a multicenter study. Hepatol Commun 7:e0073. 10.1097/HC9.0000000000000073 36881615 10.1097/HC9.0000000000000073 PMC9995094

[17] Koo E, Seif El Dahan K, Daher D et al (2024) Risk of hepatocellular carcinoma in subcentimeter liver nodules identified on surveillance ultrasound: a systematic review. Clin Gastroenterol Hepatol 23:1320–1327.e2. 10.1016/j.cgh.2024.08.051 39481466 10.1016/j.cgh.2024.08.051

[18] Sinn DH, Yi J, Choi MS et al (2013) Incidence and risk factors for surveillance failure in patients with regular hepatocellular carcinoma surveillance. Hepatol Int 7:1010–1018. 10.1007/s12072-013-9462-z 26202030 10.1007/s12072-013-9462-z

[19] Kamaya A, Fetzer DT, Seow JH et al (2024) LI-RADS US surveillance version 2024 for surveillance of hepatocellular carcinoma: an update to the American College of Radiology US LI-RADS. Radiology 313:e240169. 10.1148/radiol.240169 39625378 10.1148/radiol.240169

[20] Huang S-C, Su T-H, Tseng T-C et al (2025) Pre-existing and new-onset metabolic dysfunctions increase cirrhosis and its complication risks in chronic hepatitis B. Am J Gastroenterol 120:401–409. 10.14309/ajg.0000000000002915 38920306 10.14309/ajg.0000000000002915

[21] Mao X, Cheung KS, Peng C et al (2023) Steatosis, HBV-related HCC, cirrhosis, and HBsAg seroclearance: a systematic review and meta-analysis. Hepatology 77:1735–1745. 10.1002/hep.32792 36111362 10.1002/hep.32792

[22] Ayada I, Li J, Brouwer WP et al (2024) Impact of chronic hepatitis B and concurrent steatosis on the risk of hepatocellular carcinoma. Hepatol Int 18:1053–1055. 10.1007/s12072-024-10639-9 38451406 10.1007/s12072-024-10639-9

[23] Kim DS, Jeon MY, Lee HW et al (2019) Influence of hepatic steatosis on the outcomes of patients with chronic hepatitis B treated with entecavir and tenofovir. Clin Mol Hepatol 25:283–293. 10.3350/cmh.2018.0054 30419649 10.3350/cmh.2018.0054 PMC6759433

[24] Huang S-C, Su T-H, Tseng T-C et al (2023) Distinct effects of hepatic steatosis and metabolic dysfunction on the risk of hepatocellular carcinoma in chronic hepatitis B. Hepatol Int 17:1139–1149. 10.1007/s12072-023-10545-6 37247045 10.1007/s12072-023-10545-6

[25] Cho H, Chang Y, Lee J-H et al (2020) Radiologic nonalcoholic fatty liver disease increases the risk of hepatocellular carcinoma in patients with suppressed chronic hepatitis B. J Clin Gastroenterol 54:633–641. 10.1097/MCG.0000000000001217 31033805 10.1097/MCG.0000000000001217

[26] Kim MN, Han K, Yoo J et al (2022) Increased risk of hepatocellular carcinoma and mortality in chronic viral hepatitis with concurrent fatty liver. Aliment Pharmacol Ther 55:97–107. 10.1111/apt.16706 34820871 10.1111/apt.16706

[27] Lee YB, Ha Y, Chon YE et al (2019) Association between hepatic steatosis and the development of hepatocellular carcinoma in patients with chronic hepatitis B. Clin Mol Hepatol 25:52–64. 10.3350/cmh.2018.0040 30360031 10.3350/cmh.2018.0040 PMC6435969

[28] Peleg N, Issachar A, Sneh Arbib O et al (2019) Liver steatosis is a strong predictor of mortality and cancer in chronic hepatitis B regardless of viral load. JHEP Rep 1:9–16. 10.1016/j.jhepr.2019.02.002 32039349 10.1016/j.jhepr.2019.02.002 PMC7001543

[29] Oh JH, Lee HW, Sinn DH et al (2021) Controlled attenuation parameter value and the risk of hepatocellular carcinoma in chronic hepatitis B patients under antiviral therapy. Hepatol Int 15:892–900. 10.1007/s12072-021-10205-7 34260013 10.1007/s12072-021-10205-7

[30] Mak L-Y, Hui RW-H, Fung J et al (2021) Reduced hepatic steatosis is associated with higher risk of hepatocellular carcinoma in chronic hepatitis B infection. Hepatol Int 15:901–911. 10.1007/s12072-021-10218-2 34152534 10.1007/s12072-021-10218-2

[31] Huang S-C, Su T-H, Tseng T-C et al (2025) All-cause and cause-specific mortality in patients with chronic hepatitis B and concurrent steatotic liver disease. J Hepatol 83:43–51. 10.1016/j.jhep.2024.12.009 39675434 10.1016/j.jhep.2024.12.009

[32] Lockart I, Yeo MGH, Hajarizadeh B et al (2022) HCC incidence after hepatitis C cure among patients with advanced fibrosis or cirrhosis: a meta-analysis. Hepatology 76:139–154. 10.1002/hep.32341 35030279 10.1002/hep.32341 PMC9303770

[33] Pol S, ANRS/AFEF Study Group (2021) Similar 5-year HCC occurrence in Tenofovir- and Entecavir-treated HBV chronic infection in the French AFEF/ANRS CO22 Hepather cohort. Aliment Pharmacol Ther 53:616–629. 10.1111/apt.16197 10.1111/apt.16197 33464621

[34] Hwang JA, Kang TW, Min JH et al (2022) Association between intensity of imaging surveillance and clinical outcomes in patients with hepatocellular carcinoma. Eur J Radiol 151:110328. 10.1016/j.ejrad.2022.110328 35489206 10.1016/j.ejrad.2022.110328

[35] Barr RG, Wilson SR, Rubens D et al (2020) Update to the Society of Radiologists in ultrasound liver elastography consensus statement. Radiology 296:263–274. 10.1148/radiol.2020192437 32515681 10.1148/radiol.2020192437

