# Supplementary material for: Hepatocellular carcinoma risk stratification to identify patients suitable for intensive surveillance in viral hepatitis: the SELECT score
Source: Eur Radiol. 2025 Oct 25;36(4):2929–42. doi: 10.1007/s00330-025-12060-8 (PMC13035526; doi:10.1007/s00330-025-12060-8)

Hepatocellular Carcinoma Risk Stratification to Identify Patients Suitable for Intensive Surveillance in Viral Hepatitis: the SELECT score

ELECTRONIC SUPPLEMENTARY MATERIAL

Hepatocellular carcinoma (HCC) risk scoring systems

aMAP risk score— The age-male-albumin-bilirubin-platelets (aMAP) score is calculated based on following equation [1].

$$\text{aMAP} = (\{0.06 \times \text{age} + 0.89 \times \text{sex} + 0.48 \times [(\log_{10} \text{bilirubin} \times 0.66) + (\text{albumin} \times -0.085)] - 0.01 \times \text{platelets}\} + 7.4) / 14.77 \times 100$$

where age is in years, sex is 0 for female, 1 for male, bilirubin has units of μmol/L, albumin is expressed as g/L, and platelets are represented as $10^3/mm^3$. The high-risk group (aMAP score 60–100) had an annual HCC incidence of 1.6–4.0% [1].

Toronto HCC risk index— The Toronto HCC Risk Index (THRI) accounts for age, etiology, gender and platelets counts for HCC risk prediction [2].

$$\text{THRI} = \text{Age score} + \text{Etiology score} + \text{Gender score} + \text{Platelet score}$$

Each category of age, etiology, gender and platelet has its own scoring system as follows: a) age (0 in less than 45 years, 50 for age between 45 and 60 years, 100 for over 60 years); b) etiology (0 for autoimmune hepatitis and sustained virological response of hepatitis C virus, 54 for steatohepatitis, 97 for hepatitis B virus and hepatitis C virus, and 36 for others); c) sex (0 for female, 80 for male); d) platelets (0 for more than 200 [x 10^9/L], 20 for platelet count between 140 and 200 [x 10^9/L], 70 for platelet count between 80 and 139 [× 10^9/L], and 89 for platelet count less than 80 [× 10^9/L]. The high-risk group (THRI > 240) had a 10-year cumulative HCC incidence of 32%, while the low-risk (THRI <120) and medium-risk (120–240)

groups had 3% and 10% 10-year cumulative HCC incidence rates, respectively [2].

ADRESS-HCC score—The ADRESS (Age, Diabetes, Race, Etiology of cirrhosis, Sex, and Severity)-HCC score is calculated based on following equation [3]:

$$\text{ADRESS-HCC} = \text{Age} \times 0.0532 + \text{Diabetes} \times 0.2135 + \text{Race} \times 0.2058 + \text{Etiology} + \text{Sex} \times 0.5114 + \text{Severity} \times 0.1170$$

with the variables coded as follows: age (years), diabetes (0 for absence, 1 for presence), race (0 for non-Hispanic white, 1 for nonwhite or Hispanic), etiology (0 for autoimmune, 0.359 for alcohol or metabolic liver disease, 1.246 for viral hepatitis), sex (0 for female, 1 for male), and severity (Child-Pugh score: from 5 to 15). In the external cohort with Child-Pugh class A, the high-risk group (score of 4.71) had a hazard ratio of 6.9 (95% CI: 1.9–43.9) [3].

Velazquez score— The Velazquez score considers age, platelet, serology (hepatitis C virus) and prothrombin time, and is calculated based on following equation [4]:

$$\text{Velazquez score} = 1.65\ (\text{prothrombin activity} \leq 75\%) + 1.41\ (\text{age} \geq 55 \text{ years}),\ 0.92\ (\text{platelet count} < 75\ [\times 10^3/\text{mm}^3]) + 0.74\ (\text{if hepatitis C virus antibody positive})$$

where the prothrombin activity is expressed as a percentage and age in years. The high-risk group (score >2.33) had a 4-year cumulative HCC incidence of 30.1%.

Modified PAGE-B—The modified PAGE-B score is calculated by summing the scores for age, gender, platelet counts and albumin level, and each category has scoring system [5].

$$\text{Modified PAGE-B score} = \text{Age score} + \text{Gender score} + \text{Platelet score} + \text{Albumin score}$$

The age score is from 0 for 11 (0 for less than 30 years, 3 for 30–39 years, 5 for 40–49 years, 7 for 50–59 years, 9 for 60–69 years and 11 for 70 years or older). In the gender score, 2

points and 0 points are assigned to male and female, respectively. The platelet score is categorized into five levels based on the platelet count (× 10^9/L). A score of 0 is assigned for platelet counts ≥ 250, while a score of 5 is given for platelet counts < 100. For platelet counts between 200 and 250, a score of 2 is assigned. The score increases by 1 point for every 50-platelet count decrease until it reaches < 100. The albumin score is between 0 and 3 according to following category: 0 for serum albumin level (g/dL) of ≥ 4.0, 1 for 3.5–4.0, 2 for 3–3.5 and 3 for <3. The high-risk group (≥13) had a 5-year cumulative incidence of 18.2% in a validation group [5].

HCC diagnosis

The presence or absence of HCC was determined based on the diagnosis in electronic medical chart database of each institution (International Classification of Diseases, 10th Revision [ICD-10] code C22.0; or Korean Standard Classification of Diseases, KCD code 22.0 which was based on ICD-10). In three institutions, HCC diagnosis was primarily made based on imaging features including arterial phase hyperenhancement (APHE) and portal or delayed washout on contrast-enhanced CT and dynamic MRI using extracellular contrast agent. In patients with gadoxetic acid-enhanced MRI which was readily used after 2008 in South Korea, APHE and portal washout or transitional phase hypointensity and hepatobiliary phase hypointensity were used to diagnose HCC. In patients with dual-agent MRI using extracellular contrast media and superparamagnetic iron oxide (SPIO) agent, being used until 2010 in South Korea, HCC was defined as observations with APHE and hyperintensity on post-contrast T2 or T2* images compared with liver parenchyma. Hepatic observations with APHE on CT and MRI but no portal or delayed washout, those underwent SPIO-enhanced MRI and regarded as HCC if the observations showed hyperintensity on post-contrast T2 or T2* images.

US feature review for inter-observer agreement

Cirrhosis on US was determined based on the following criteria: liver segmental imbalance, surface nodularity, accentuation of hepatic fissures, markedly coarse echogenicity with decreased penetration, presence of cirrhotic nodules, and evidence of portal hypertension [6]. Multiple cirrhotic nodules were defined as the presence of five or more discrete or ill-defined hyper- or hypo-echoic nodules in similar sizes (<20 mm) [6–8].

Statistical analysis

Development of the prediction model—The proportional hazard assumption was checked using restricted cubic splines for continuous predictors and log-log survival plots for categorical predictors. The restricted cubic spline method was used to check the assumption of linearity for continuous predictors. The variables of age, sex, alcohol intake, serum albumin, total bilirubin, platelet count, alanine transaminase, prothrombin time, diabetes, presence of chronic liver disease, alcohol intake, cirrhosis, hepatic steatosis, cirrhotic nodules, and splenomegaly on US were considered for the prediction models. The predictors for the prediction model were selected using the variable selection method to address potential multicollinearity. Variance inflation factors (VIFs) were also calculated, and all VIFs in the final model were below 1.2. Backward elimination, forward selection and the best subset selection produced consistent results, supporting the stability of the selected predictors. Eight predictors were ultimately selected for the prediction model. Therefore, all possible two-way interactions among them were tested using a Bonferroni-adjusted significance level of 0.002 (i.e., 0.05 divided by the number of pairwise Combinations, 8C2) to reduce the risk of false-positive results from interaction testing.

Cut-offs for three-group stratification—To distinguish low-risk and high-risk groups, we

calculated the pair of two thresholds that would maximize the difference in survival curves among the three groups defined by the two arbitrary scores (cut-off for low-risk and cut-off for high-risk) in the scores calculated from the prediction model (SELECT). The cut-off values that maximized the difference in survival curves were determined based on the significance of the log-rank test results for the survival curves of the three groups and were analyzed using an x-tile plot in the development dataset. The calculated pair of two cut-offs were applied to the internal and external validation datasets to calculate the predicted HCC incidence.

Cut-offs for identifying groups with annual HCC incidence of 2.5% or higher— In the development dataset, after the prediction model was finalized, a post hoc cut-off value of >-2.04 was selected; this cut-off corresponded to an estimated 5-year HCC incidence of 12.5%, assuming a constant HCC incidence of 2.5% annually. No optimization was performed for a specific clinical cut-off during the model estimation process, and risk categories were defined post hoc to facilitate clinical interpretation after model finalization.

Sensitivity and specificity of the model—In high-risk group, 5-year sensitivity refers to the proportion of patients classified as high-risk among those who developed HCC within 5 years. The 5-year specificity is the proportion of patients not classified as high-risk among those who did not develop HCC within 5 years. Both sensitivity and specificity are time-dependent. For comparison between sensitivity and specificity among the risk stratification systems, we used the McNemar test and showed adjusted *P*-values using the step-down Bonferroni method for pairwise comparisons.

SUPPLEMENTARY RESULTS

Presence of hepatic steatosis according to liver disease severity

On US, hepatic steatosis was observed in 24.3% (4273/17557) patients. In patients with hepatic steatosis, 58.6% (2503), 34.7% (1489) and 6.8% (289) patients showed normal echogenicity which referred absence of chronic liver disease or cirrhosis, chronic liver disease and cirrhosis on US. The findings were consistent in development, internal validation and external validation datasets. In development dataset, 29.3% (2321/7918) had steatosis on US, and those showed normal echogenicity in 52.2% (1212), chronic liver disease in 40.5% (941) and cirrhosis in 7.2% (168). In Internal validation dataset, 28.8% (978/3393) revealed steatosis on US: 48.0% (469), 44.8% (438) and 7.3% (71) patients had normal echogenicity, chronic liver disease and cirrhosis, respectively. In external validation dataset, 15.6% (974/6246) had steatosis. Among 974 patients with steatosis, 84.4% (822) had normal echogenicity while 10.5% (102) and 5.1% (50) showed accompanying chronic liver disease and cirrhosis, respectively.

Antiviral medication in the study population

Antiviral medications were administered in 16.7% (1324/7918), 18.1% (613/3393) and 7.7% (478/6246) of the development, internal validation, and external validation datasets, respectively, at the time of index US. In the development dataset, among the patients on anti-viral medications (n = 1324), the following were used: entecavir (n = 493), lamivudine (n = 337), tenofovir (n = 162), adefovir (n = 153), telbivudine (n = 72), and the combination of zidovudine and lamivudine (n = 2) for hepatitis B, and ribavirin (n = 105) for hepatitis C. In the internal validation dataset, among those anti-viral medications (n = 613), the medications included entecavir (n = 210), lamivudine (n = 165), tenofovir (n = 83), adefovir (n = 66), telbivudine (n = 24) and the combination of zidovudine and lamivudine (n = 2) for hepatitis B and ribavirin (n = 63) for hepatitis C. In the external validation dataset, anti-viral medications (n = 478) included adefovir (n = 217), lamivudine (n = 94), entecavir (n = 70), ribavirin (n = 63),

tenofovir (n = 16), telbivudine (n = 4), clevudine/adefovir (n = 2), pradefovir (n = 2), sofosbuvir/ledipasvir (n = 2), lamivudine/abacavir (n = 1), zidovudine/lamivudine (n = 1), emtricitabine/tenofovir (n = 1), ritonavir (n = 1), sofosbuvir (n = 1), danoprevir/ritonavir (n = 1) and daclatasvir/asunaprevir (n = 1).

Interaction between variables in model development

Interaction between platelet counts and cirrhosis on US—Our multivariable analysis revealed a significant interaction between platelet count and US-defined cirrhosis in predicting HCC development (*P* <0.001). This indicates that the effect of each variable on HCC risk is modified by the status of the other. For example, the prognostic impact of US-defined cirrhosis was not constant; its hazard ratio (HR) was substantially higher (3.661 [95% CI: 3.026, 4.429] vs. 2.184 [95% CI: 1.918, 2.481]) in patients with normal platelet counts (221 $\times 10^9$/L, corresponding 3rd quartile in development dataset) compared to those with low platelet counts (135 $\times 10^9$/L, corresponding 1st quartile in development dataset). Conversely, the influence of platelet count on HCC risk was also dependent on the presence of cirrhosis. Given the statistical significance of this interplay, the interaction term was incorporated into the final SELECT model to accurately estimate HCC risk.

Significance of steatosis on US—To further explore the low HR of hepatic steatosis in unadjusted univariable analysis, we calculated the HR of hepatic steatosis after adjusting liver parenchymal echogenicity on US. When adjusting liver parenchymal echogenicity, the impact of hepatic steatosis became insignificant (HR: 0.916 [95% CI: 0.791, 1.061], *P* = 0.242) while liver cirrhosis on US still held significantly higher HR compared to non-cirrhosis after adjusting hepatic steatosis. There was no significant interaction between liver parenchymal echogenicity and hepatic steatosis.

Demographics of 217 patients in the inter-observer agreement US image review session

US images of 217 patients (male = 97; mean age 54.3 years [95% CI: 53.2, 55.4]) were retrospectively reviewed. Approximately 83.9% (182/217) had hepatitis B, followed by hepatitis C (14.7%, 32/217) and co-infection of hepatitis B and C (1.4%, 3/217), and 20.7% (45/217) had diabetes. In original US reports, cirrhosis was observed in 24% (52/217), and multiple cirrhotic nodules were observed in 9.2% (20/217). Hepatic steatosis was reported in 33.6% (73/217).

SUPPLEMENTARY REFERENCES

1. Fan R, Papatheodoridis G, Sun J, et al (2020) aMAP risk score predicts hepatocellular carcinoma development in patients with chronic hepatitis. J Hepatol 73:1368–1378. https://doi.org/10.1016/j.jhep.2020.07.025

2. Sharma SA, Kowgier M, Hansen BE, et al (2018) Toronto HCC risk index: A validated scoring system to predict 10-year risk of HCC in patients with cirrhosis. J Hepatol 68:92–99. https://doi.org/10.1016/j.jhep.2017.07.033

3. Flemming JA, Yang JD, Vittinghoff E, et al (2014) Risk prediction of hepatocellular carcinoma in patients with cirrhosis: The ADRESS-HCC risk model. Cancer 120:3485–3493. https://doi.org/10.1002/cncr.28832

4. Velázquez RF, Rodríguez M, Navascués CA, et al (2003) Prospective analysis of risk factors for hepatocellular carcinoma in patients with liver cirrhosis. Hepatology 37:520–527. https://doi.org/10.1053/jhep.2003.50093

5. Kim JH, Kim YD, Lee M, et al (2018) Modified PAGE-B score predicts the risk of hepatocellular carcinoma in Asians with chronic hepatitis B on antiviral therapy. J Hepatol 69:1066–1073. https://doi.org/10.1016/j.jhep.2018.07.018

6. Aubé C, Bazeries P, Lebigot J, et al (2017) Liver fibrosis, cirrhosis, and cirrhosis-related nodules: Imaging diagnosis and surveillance. Diagn Interv Imaging 98:455–468. https://doi.org/10.1016/j.diii.2017.03.003

7. Kanematsu M, Hoshi H, Yamada T, et al (1999) Small hepatic nodules in cirrhosis: ultrasonographic, CT, and MR imaging findings. Abdom Imaging 24:47–55. https://doi.org/10.1007/s002619900439

8. Jang H-J, Kim TK, Wilson SR (2009) Small nodules (1-2 cm) in liver cirrhosis: characterization with contrast-enhanced ultrasound. Eur J Radiol 72:418–424. https://doi.org/10.1016/j.ejrad.2008.08.011

Table S1. Comparison of multivariable Cox regression analysis between the SELECT model and a clinical model without ultrasound features in the development dataset

	SELECT Model		Clinical Model	
**Variables**	**HR (95% CI)**	***P*-value**	**HR (95% CI)**	***P*-value**
Age (per year)	1.028 (1.021, 1.034)	<0.001	1.024 (1.017, 1.03)	<0.001
Male sex	1.714 (1.524, 1.926)	<0.001	1.831 (1.629, 2.057)	<0.001
Diabetes mellitus (yes)	1.153 (1.025, 1.296)	0.02	1.156 (1.028, 1.3)	0.016
Albumin (per g/dL)	0.429 (0.370, 0.497)	<0.001	0.408 (0.353, 0.472)	<0.001
ALT (per U/L)	1.001 (1.000, 1.002)	0.006	1.001 (1, 1.001)	0.049
Platelet (per 10^9/L)			0.992 (0.991, 0.992)	<0.001
Cirrhosis on US (no)	0.992 (0.991, 0.994)	<0.001	–	–
Cirrhosis on US (yes)	0.998 (0.997, 0.999)	<0.001	–	–
Cirrhosis on US†			–	–
Platelet count = 100 (per 10^9/L)	1.770 (1.512, 2.072)	<0.001	–	–
Platelet count = 150 (per 10^9/L)	2.390 (2.100, 2.715)	<0.001	–	–
Platelet count = 200 (per 10^9/L)	3.228 (2.744, 3.798)	<0.001	–	–
Multiple cirrhotic nodules (yes)	1.398 (1.206, 1.620)	<0.001	–	–

Table S2. Comparison of SELECT model with clinical model excluding ultrasound features

Dataset	Uno C-index			
	SELECT	Clinical model	Difference	*P*-value
**Internal validation**	0.747 (0.726, 0.767)	0.721 (0.702, 0.741)	0.026 (0.013, 0.038)	<0.001
**External validation**	0.791 (0.764, 0.819)	0.765 (0.734, 0.795)	0.027 (0.013, 0.04)	<0.001

Note—.: Clinical model refers to a Cox regression model utilizing only clinical variables in Table S1.

Table S3. Cumulative incidence of HCC according to the SELECT score in each dataset

Time (y)	Development dataset (n = 7918)			Internal validation dataset (n = 3393)			External validation dataset (n = 6246)		
	Low	Intermediate	High	Low	Intermediate	High	Low	Intermediate	High
Incidence	7.4 (365/4907)	23.6 (386/1633)	44.3 (611/1378)	7.4 (155/2101)	24.3 (171/702)	42.5 (251/590)	2.4 (100/4153)	13.4 (159/1184)	25.9 (235/909)
1 y	0.02 [0, 0.06]	0.06 [0, 0.18]	0.07 [0, 0.21]	0.05 [0, 0.14]	0.14 [0, 0.42]	0.17 [0, 0.50]	0.0 [0, 0]	0.0 [0, 0]	0.10 [0, 0.30]
3 y	0.66 [.43, .89]	3.68 [2.74, 4.62]	11.64 [9.90, 13.38]	0.04 [0.60, 1.48]	3.13 [1.81, 4.45]	12.87 [10.07, 15.67]	0.40 [0.20, 0.60]	1.60 [0.90, 2.30]	8.00 [6.20, 9.90]
5 y	2.06 [1.64, 2.48]	9.98 [8.45, 11.51]	24.83 [22.42, 27.24]	2.17 [1.52, 2.82]	7.95 [5.85, 0.05]	25.97 [22.23, 29.71]	0.80 [0.50, 1.10]	6.90 [5.40, 8.40]	16.1 [13.60, 18.60]
10 y	6.36 [5.56, 7.16]	22.47 [20.16, 24.78]	48.11 [45.05, 51.17]	6.89 [5.61, 8.17]	25.7 [21.86, 29.54]	44.15 [39.56, 48.74]	2.70 [2.10, 3.30]	16.3 [13.60, 19.00]	31.5 [27.70, 35.30]
15 y	14.16 [12.61, 15.71]	36.02 [32.77, 39.27]	60.76 [57.21, 64.31]	13.11 [10.95, 15.27]	37.75 [32.71, 42.79]	60.05 [54.07, 66.03]	4.50 [3.50, 5.60]	23.2 [19.10, 27.30]	41.5 [35.60, 47.50]

HR	1	3.414 [2.959, 3.939]	8.136 [7.145, 9.265]	1	3.566 [2.869, 4.432]	8.117 [6.64, 9.922]	1	5.93 [4.617,7.616]	13.038 [10.313,16.484]

Note—. Values are percentages (numerators/denominators) or percentages [95% confidence intervals]. Incidence is presented as patients who developed HCC/total number of patients in each group. HCC = hepatocellular carcinoma; HR = hazard ratio.

Table S4. Cumulative incidence of HCC in patients with SELECT score ≤-2.04 in each dataset

Variables	Development dataset (n = 7918)	Internal validation dataset (n = 3393)	External validation dataset (n = 6246)
Number of patients with SELECT score <=- 2.04	80.0 (6336)	80.3 (2726)	82.1 (5130)
5-year HCC cumulative incidence (%)	3.7 [3.2, 4.2]	3.4 [2.6, 4.1]	1.9 [1.5, 2.3]
10-year HCC cumulative incidence (%)	10 [9.1, 10.8]	10.8 [9.4, 12.2]	5.4 [4.6, 6.2]

Note—. Values are percentages (absolute numbers) unless otherwise specified. Numbers in brackets are 95% CI. HCC = hepatocellular carcinoma.

Table S5. Performance of risk scores for predicting HCC development in hepatitis B patients in the external validation dataset.

**Variables (n = 5112)**	**SELECT**	**aMAP**	**THRI**	**ADRESS-HCC**	**Velazquez et al.**	**mPAGE-B**
Cut-offs	>-2.04	≥60	>240	≥4.71	>2.33	≥13
Number of patients*	17.8 (910/5112)	71.1 (3634/5112)	20.4 (1043/5112)	81.2 (4152/5112)	8.7 (444/5112)	22.9 (1171/5112)
5-year cumulative incidence (%)	14.6 [12.2, 17]	5.6 [4.8, 6.4]	7.3 [5.6, 8.9]	4.9 [4.2, 5.6]	14.5 [11.1, 17.9]	10.3 [8.5, 12.1]
10-year cumulative incidence (%)	28.1 [24.6, 31.7]	12.1 [10.8, 13.4]	16 [13.4, 18.7]	10.8 [9.7, 12]	25.9 [20.9, 30.9]	21.7 [18.7, 24.7]
Sensitivity (%)†	63.0 (123/196) [56.0, 70.0]	95.0 (187/196) [91.0, 98.0]	36.0 (70/196) [29.0, 43.0]	95.0 (186/196) [91.0, 98.0]	31.0 (60/196) [24.0, 38.0]	56.0 (110/196) [49.0, 63.0]
P-value‡	Ref	<0.001	<0.001	<0.001	<0.001	0.08
Specificity (%)†	84.0 (4129/4916) [83.0, 85.0]	30.0 (1469/4916) [29.0, 31.0]	80.0 (3943/4916) [79.0, 81.0]	19.0 (950/4916) [18.0, 20.0]	92.0 (4532/4916) [91.0, 93.0]	78.0 (3855/4916) [77.0, 80.0]
P-value‡	Ref	<0.001	<0.001	<0.001	<0.001	<0.001
PPV (%)†	13.5 (123/910) [11.4, 15.9]	5.2 (187/3634) [4.5, 5.9]	6.7 (70/1043) [5.3, 8.4]	4.5 (186/4152) [3.9, 5.2]	13.5 (60/444) [10.5, 17.1]	9.4 (110/1171) [7.8, 11.2]
P-value‡	Ref	<0.001	<0.001	<0.001	0.998	<0.001
NPV (%)†	98.3 (4129/4202) [97.8, 98.6]	99.4 (1469/1478) [98.9, 99.7]	96.9 (3943/4069) [96.3, 97.4]	99.0 (950/960) [98.1, 99.5]	97.1 (4532/4668) [96.6, 97.6]	97.8 (3855/3941) [97.3, 98.3]

P-value‡	Ref	0.013	<0.001	0.10	<0.001	0.03
Accuracy (%)†	83.2 (4252/5112) [82.1, 84.2]	32.4 (1656/5112) [31.1, 33.7]	78.5 (4013/5112) [77.4, 79.6]	22.2 (1136/5112) [21.1, 23.4]	89.8 (4592/5112) [89, 90.6]	77.6 (3965/5112) [76.4, 78.7]
P-value‡	Ref	<0.001	<0.001	<0.001	<0.001	<0.001

Note—. Values are percentages (numerators/denominators). Numbers in brackets are 95% confidence intervals (CIs). Number of patients with values equal to or higher than the cut-offs. HCC = hepatocellular carcinoma; THRI = Toronto HCC Risk Index; PPV = positive predictive value; NPV = negative predictive value. *: incidence in patients eligible for intensive surveillance. †: for 5-year HCC development. ‡: adjusted *P*-value using the step-down Bonferroni method for comparison with SELECT.

SUPPLEMENTARY FIGURE

Figure S1. Interaction plot showing the effect of ultrasound assessed liver parenchyma on hepatocellular carcinoma (HCC) risk by platelet count level. Hazard ratios are shown as the solid line with gray shaded 95% confidence intervals. The vertical dashed line indicates the threshold representing lower normal range (150 × 10^9/L). The plot illustrates that the predictive impact of ultrasound cirrhosis on HCC risk increases with higher platelet counts, being most pronounced in patients with normal platelet levels. *CI = confidence interval HR = hazard ratio, plt = platelet count*

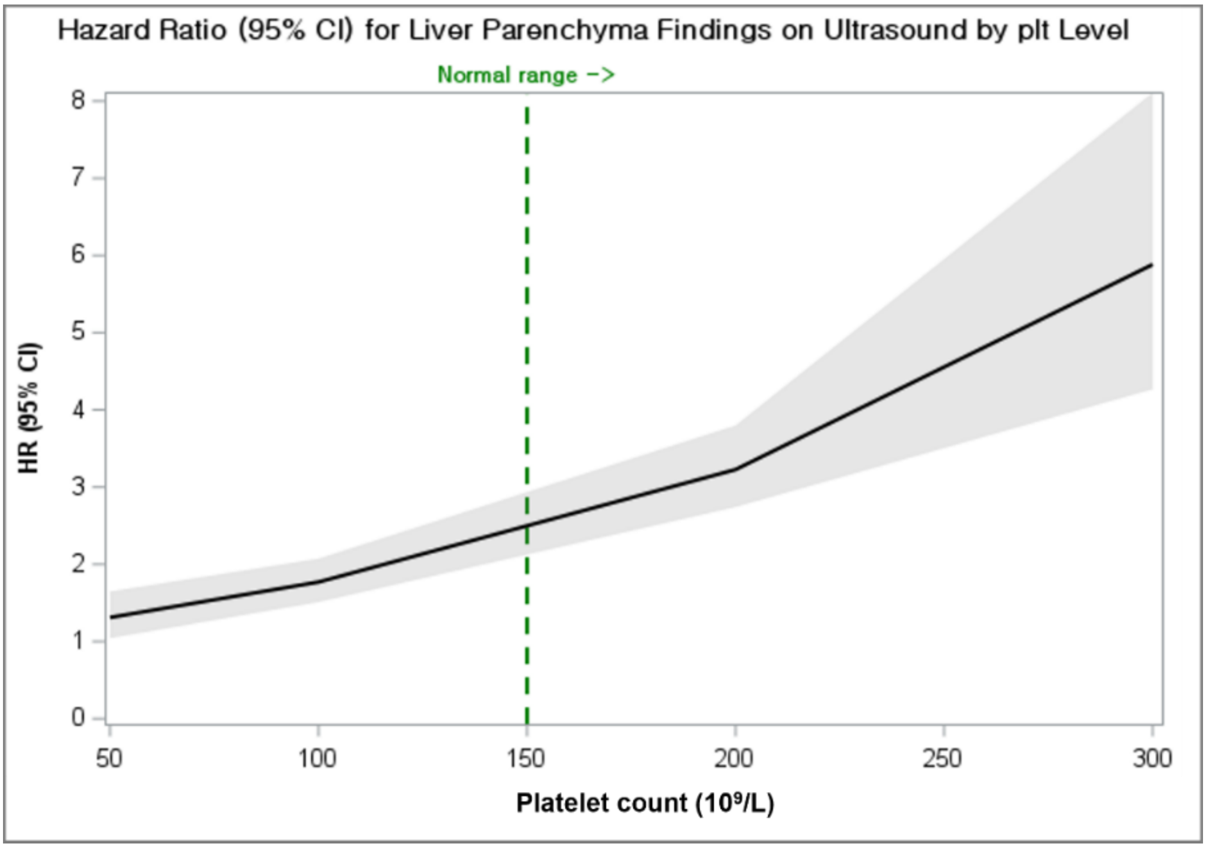

Figure S2. Calibration curves of SELECT score and Clinical model without US feature. 5-year (A) and 10-year (B) cumulative incidence of HCC internal validation dataset. Calibration curves show calibration slopes of SELECT and clinical models were 1.02 (95% CI: 0.92, 1.11) and 1.017 (95% CI: 0.916, 1.117) in internal validation dataset. 5-year (C) and 10-year (D) cumulative HCC incidence calibration curves are 1.165 (95% CI: 1.067, 1.262) and 1.196 (95% CI: 1.088, 1.304) in external validation dataset.

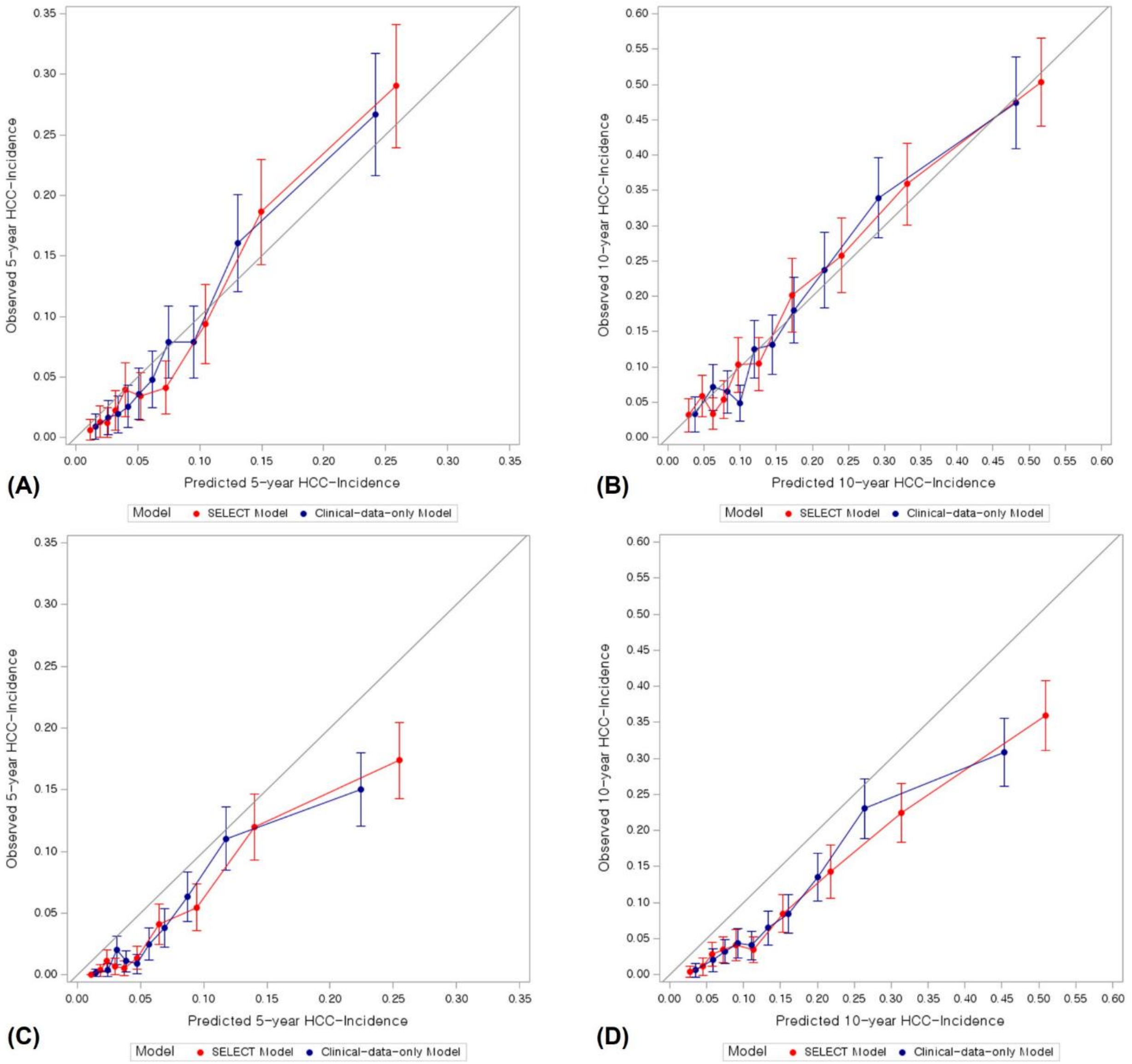

Figure S3. A 53-year-old male patient with chronic hepatitis B. The patient did not have diabetes and exhibited an albumin level of 4.1 g/dL, an alanine transaminase level of 34 U/L, and a platelet count of 232 × $10^3/mm^3$. The patient was classified as low-risk (SELECT score = -3.32). The index ultrasound (US) examination shows signs of chronic liver disease, but no cirrhosis (A). On 9-year follow-up US shows absence of hepatocellular carcinoma (B).

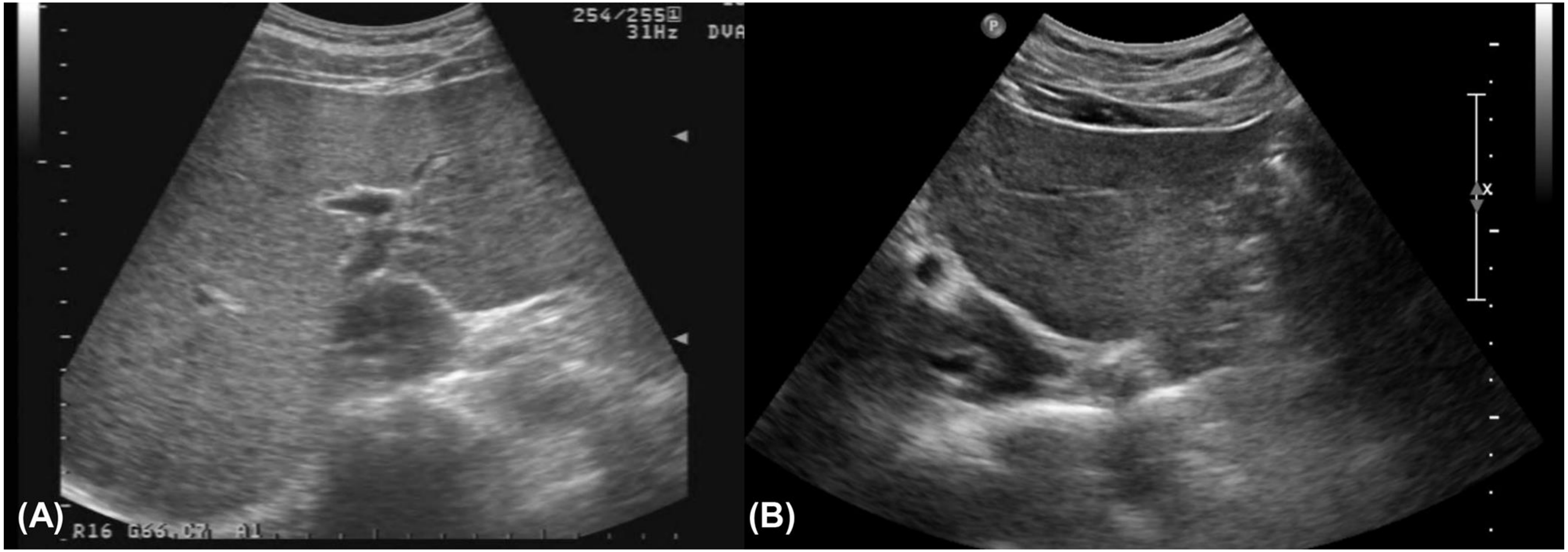

Figure S4. A 52-year-old male patient with chronic hepatitis B. The patient had diabetes, an albumin level of 4.0 g/dL, an alanine transaminase level of 19 U/L, and a platelet count of 95 × 10^3/mm^3, resulting in a high-risk classification (SELECT score = -1.51). The index ultrasound examination shows cirrhotic parenchyma (A) and 15 cm splenomegaly (B), contributing to the higher SELECT score. Follow-up computed tomography after 27 months reveals the development of ascites and HCC in segment VIII with arterial phase hyperenhancement (C, arrows) and delayed non-peripheral washout (D, arrows).

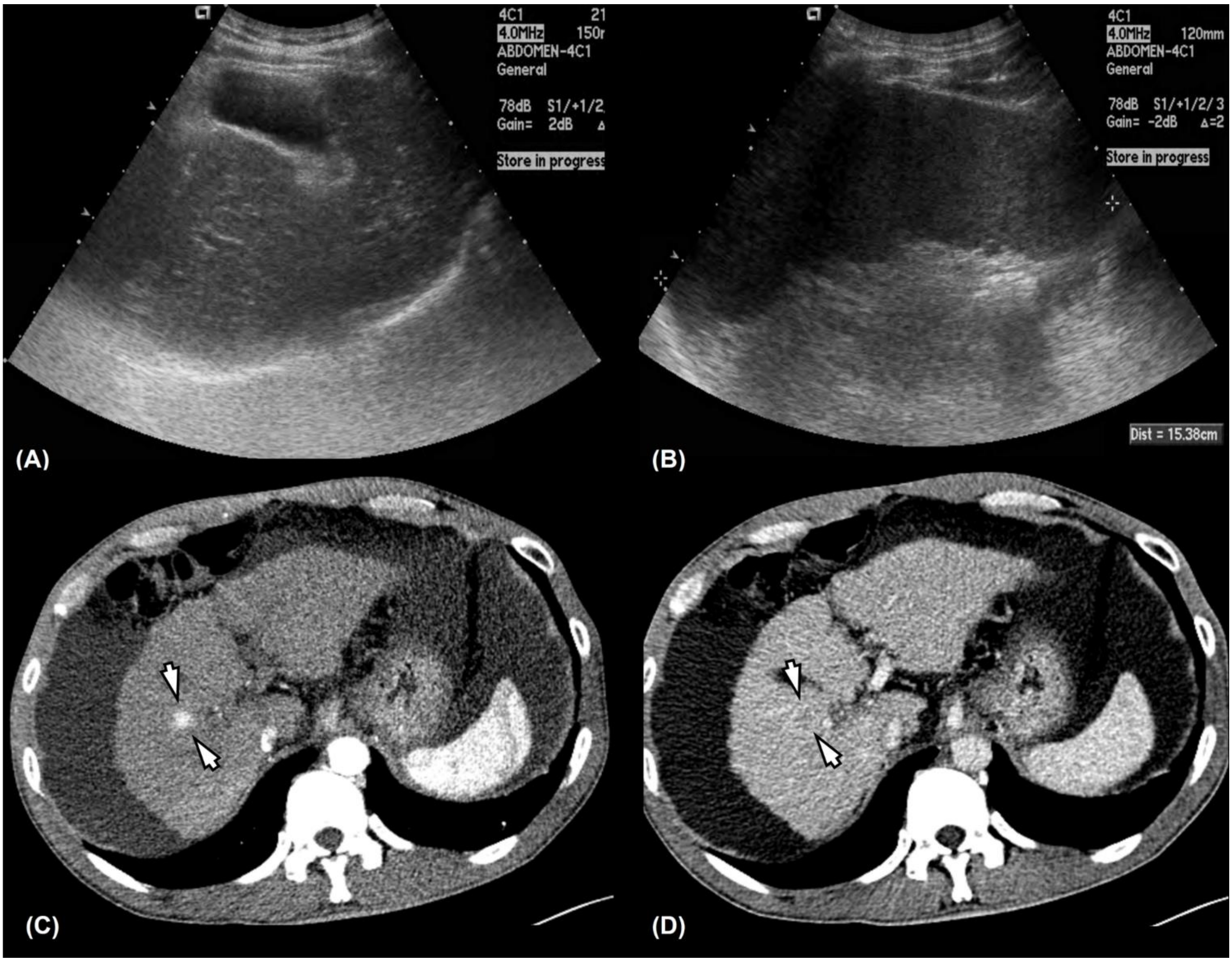

Figure S5. Calibration curves of SELECT score. 5-year (A) and 10-year (B) cumulative incidence of HCC. Calibration curves show calibration slopes of 1.02 (95% CI: 0.92, 1.11) in the internal validation dataset (red) and 1.165 (95% CI: 1.067, 1.262) in the external validation dataset (blue).

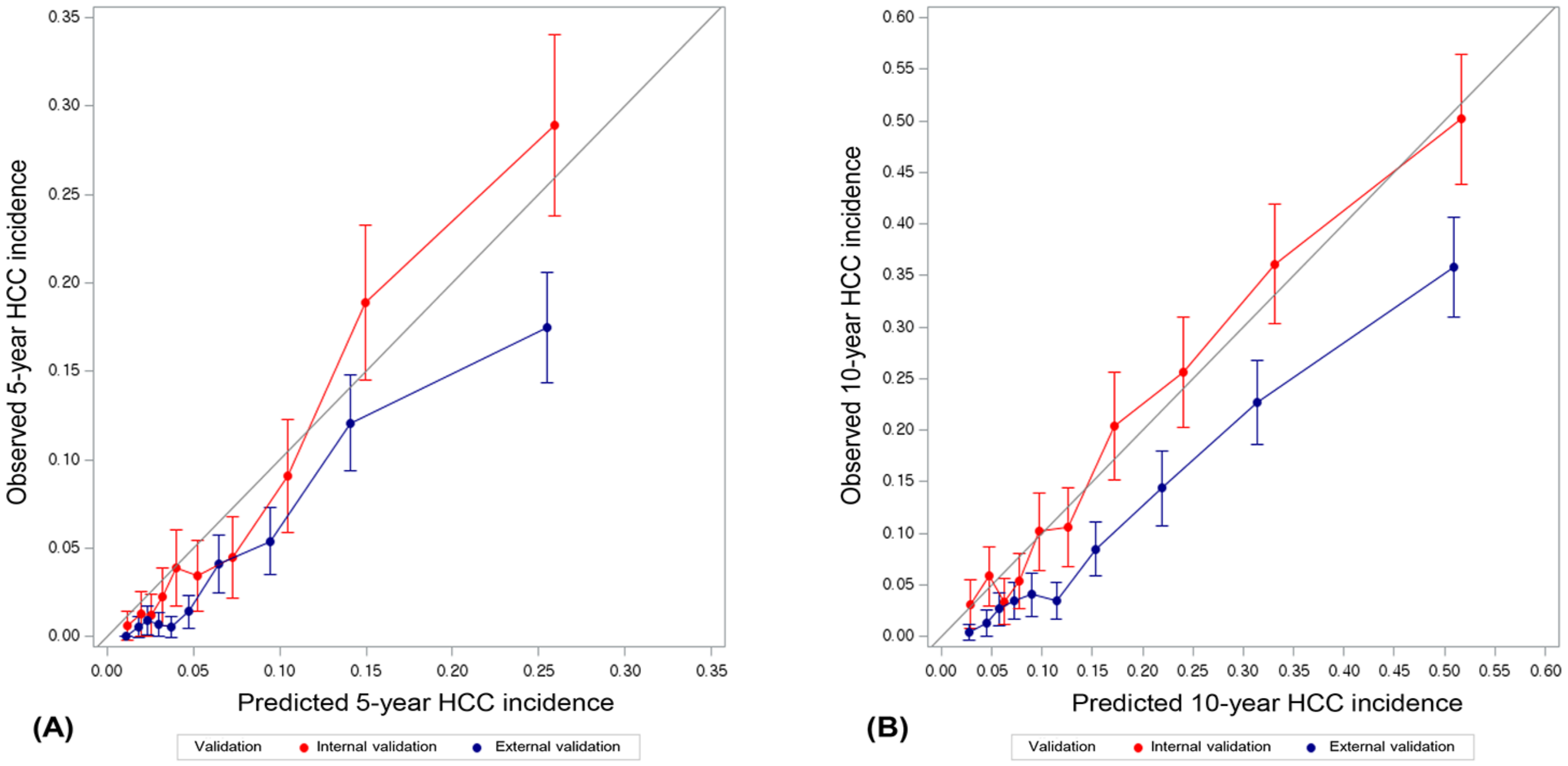

Eur Radiol (2025) Kim YY, Chang W, Lee JM, et al.

Figure S6. Liver cirrhosis on ultrasound (US). US demonstrates markedly coarse echogenicity of the liver (A) and subtle surface undulation in intercostal scan (B). Original US reported presence of cirrhosis and all four readers recorded the presence of cirrhosis.

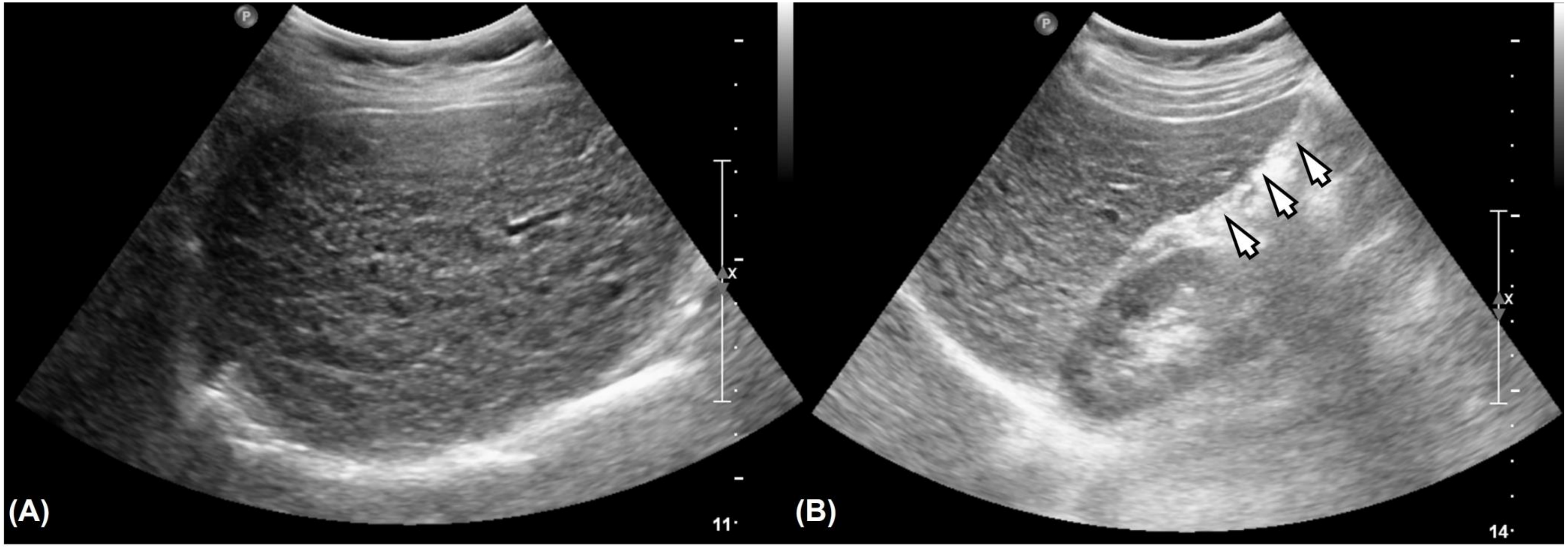

Figure S7. Multiple cirrhotic nodules on ultrasound (US). US scans at different intercostal levels reveal multiple hyperechoic nodules (arrows) in the right hemiliver (A, B). Original US reported the presence of multiple hyperechoic nodules suggestive of cirrhotic nodules. All four reviewers also reported the presence of multiple nodules in the review session.

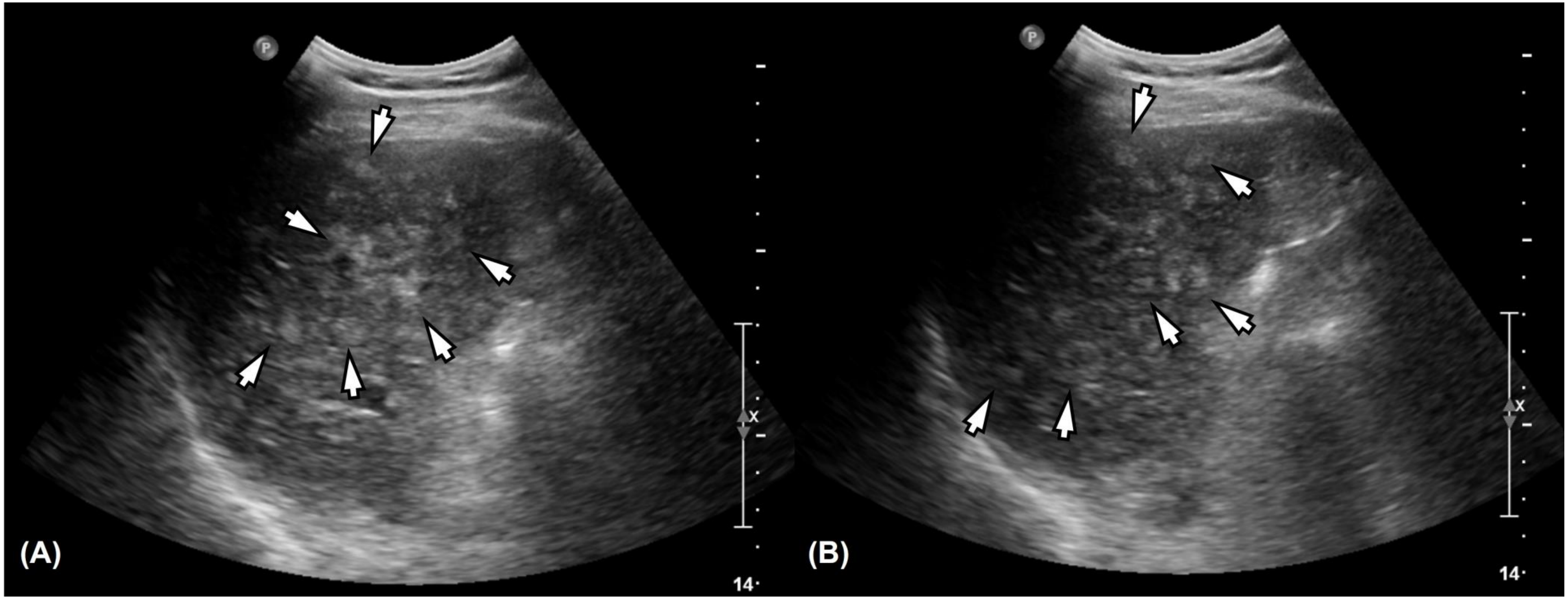

Supplement: Supplementary file 1 — Supplementary information [file 330_2025_12060_MOESM1_ESM.pdf]
